# Both a Unique Motif at the C Terminus and an N-Terminal HEAT Repeat Contribute to G-Quadruplex Binding and Origin Regulation by the Rif1 Protein

**DOI:** 10.1128/MCB.00364-18

**Published:** 2019-02-04

**Authors:** Shunsuke Kobayashi, Rino Fukatsu, Yutaka Kanoh, Naoko Kakusho, Seiji Matsumoto, Shigeru Chaen, Hisao Masai

**Affiliations:** a Department of Genome Medicine, Tokyo Metropolitan Institute of Medical Science, Kamikitazawa, Setagaya-ku, Tokyo, Japan; b Department of Biosciences, College of Humanities and Sciences, Nihon University, Tokyo, Japan; c Department of Correlative Study of Physics and Chemistry, Graduate School of Integrated Basic Sciences, Nihon University, Tokyo, Japan

**Keywords:** Cdc7/Hsk1, DNA replication, G-quadruplex, HEAT repeat

## Abstract

Rif1 is a key factor for spatiotemporal regulation of DNA replication. Rif1 suppresses origin firing in the mid-late replication domains by generating replication-suppressive chromatin architecture and by recruiting a protein phosphatase. In fission yeast, the function of Hsk1, a kinase important for origin firing, can be bypassed by *rif1*Δ due to the loss of origin suppression. Rif1 specifically binds to G-quadruplex (G4) *in vitro*. Here, we show both conserved N-terminal HEAT repeats and C-terminal nonconserved segments are required for origin suppression. The N-terminal 444 amino acids and the C-terminal 229 amino acids can each mediate specific G4 binding, although high-affinity G4 binding requires the presence of both N- and C-terminal segments. The C-terminal 91 amino acids, although not able to bind to G4, can form a multimer. Furthermore, genetic screening led to identification of two classes of *rif1* point mutations that can bypass Hsk1, one that fails to bind to chromatin and one that binds to chromatin. These results illustrate functional domains of Rif1 and indicate importance of both the N-terminal HEAT repeat segment and C-terminal G4 binding/oligomerization domain as well as other functionally unassigned segments of Rif1 in regulation of origin firing.

## Abstract (precis)

Rif1 is a key factor for spatiotemporal regulation of DNA replication. Rif1 suppresses origin firing in the mid-late replication domains by generating replication-suppressive chromatin architecture and by recruiting a protein phosphatase.

## INTRODUCTION

Spatiotemporal regulation of DNA replication permits ordered and coordinated duplication of the eukaryotic genomes (1). In higher eukaryotes, this regulation is associated with the higher-order chromosome organization in nuclei, which may be determined in early G_1_ at TDP (timing decision point) (2). The mid-late replication domains appear to be sequestered from early replicating segments both temporally and spatially, so that they will not undergo premature early replication (3).

To identify potential new regulators of replication timing, we searched for bypass mutants for *hsk1*, the gene for the fission yeast homologue of budding yeast Cdc7 kinase, known to play crucial roles in initiation of replication (4, 5). We had previously found *mrc1* mutants can weakly bypass the Hsk1 function and also had shown that replication timing is altered in *mrc1* mutants (6, 7). The screening led us to identify *rif1* as a strong bypass suppressor of the *hsk1* null mutation (8). Furthermore, the origin firing pattern was dramatically altered in *rif1*Δ cells. Following this discovery, we and others reported that Rif1 is also a major regulator of genome-wide replication timing domain structures in mammalian cells and other species as well (9–11). We proposed that Rif1 regulates replication timing through generating chromatin architecture near the nuclear periphery that is suppressive for initiation (12). Circularized chromosome conformation capture (4C) analyses indicated that Rif1 defines and restricts the interactions between replication-timing domains, supporting the role of Rif1 as an organizer of nuclear architecture (13).

Rif1, originally identified as a telomere binding factor in budding yeast (14, 15), has been shown to regulate transcriptional silencing and telomere length in concert with Rap1 (14, 16, 17). Rif1 protects telomeres through the CST (Cdc13-Stn1-Tel1) complex (18, 19) and contributes to a telomeric anticheckpoint activity (20–22). Fission yeast Rif1, through interaction with Taz1, regulates telomere length and telomere position effect (TPE) (15). *rif1*Δ restores viability of *taz1*Δ at 25°C, although the molecular mechanism of this suppression is not clear (23). Roles of Rif1 in correct telomere length regulation have been reported in other yeasts as well (24, 25). In contrast to that in yeasts, a direct role of Rif1 in telomere regulation is unlikely in mammalian cells, since it is not colocalized at telomeres and no obvious telomere defects were observed in Rif1 knockout cells (26, 27). However, Rif1 indirectly regulates telomeres through transcriptional silencing of Zscan4, a factor promoting recombination-mediated telomere elongation in embryonic stem cells (28).

In human cells, Rif1 was found to accumulate at double-stranded DNA breaks (DSBs) in an ATM- and 53BP1-dependent manner (26, 27). Rif1 also may participate in the intra-S-phase checkpoint to slow down DNA synthesis in response to DNA damage and also contributes to replication stress survival (27, 29). More recently, Rif1, in conjunction with 53BP1, was shown to facilitate nonhomologous end joining (NHEJ) repair of DSBs by inhibiting 5′ to 3′ DNA end resection required for homologous recombination (HR)-mediated repair (30–34). Yeast Rif1, on the other hand, was shown to promote DSB resection, facilitating HR repair of DSBs (35). In *Xenopus* egg extracts, Rif1 is required for chromatin accumulation of several key checkpoint proteins (TopBP1, ATR, and the MRN complex) (36). Rif1 was also reported to function in resolution of nontelomeric chromosomal entanglement in M phase in fission yeast (37).

Only limited information is available as to how the structure of Rif1 and its biochemical functions are related to its diverse cellular functions. One of the conserved features of Rif1 is the presence of HEAT (Huntingtin, elongation factor 3, A subunit of protein phosphatase 2A, and TOR) or armadillo-type helical repeats (26). The N-terminal domain of Rif1 is predicted to have 14 to 21 tandem HEAT-like repeats (29), although the numbers and lengths of HEAT-like repeats depend on the species. Within the HEAT repeats, highly conserved Rif1-core segments have been identified. In fission yeast Rif1, an ∼400-amino-acid (aa) segment (aa ~100 to ~500) is the most conserved among various HEAT repeats from many proteins, including Importin. Another conserved motif is the docking motif for type I protein phosphatase (PP1), consisting of SILK-RVXF sequences in multicellular organisms (38). In yeast, a PP1 binding motif can be found as an RVXF-SILK arrangement near the N terminus of the protein. The PP1-interacting motif was shown to be required for origin suppression activity both in yeasts and human (39–43).

It was reported that a C-terminal domain of Rif1 can bind preferentially to branched DNAs, including cruciform and forked DNA (29). It was also reported that a short segment of the C-terminal 60 amino acids (residues 1857 to 1916) of Saccharomyces cerevisiae Rif1 (ScRif1) forms a tetramer (44). We have reported that fission yeast Rif1 binds to specific segments on the chromosome (Rif1BS, for Rif1 binding site), whose sequences are characterized by the presence of multiple G-tracts, and that this binding is mediated by its specific recognition of G-quadruplex (G4) structure (45). The purified Rif1 protein binds to G4 with higher affinity than to cruciform DNA. We also purified the mouse Rif1 protein and showed that it also selectively and preferentially bound to G4 (46).

In order to elucidate how the functional domains on Rif1 protein are coordinated in its cellular activities, we genetically and biochemically characterized the truncated polypeptides of fission yeast Rif1 and also isolated loss-of-function mutants. Our results indicate the importance of both N-terminal and C-terminal domains for origin suppression and telomere regulation functions of Rif1 and requirement of their coordination for its *in vivo* chromatin binding and biological functions. Our results also identify critical residues for *in vivo* chromatin binding and origin suppression/telomere regulation. On the basis of biochemical properties of Rif1 and its truncation polypeptides, we will speculate on how it may contribute to the formation of replication-inhibitory chromatin architecture.

## RESULTS

### Both N-terminal and C-terminal segments of fission yeast Rif1 are required for its origin suppression activity.

**FIG 1:**
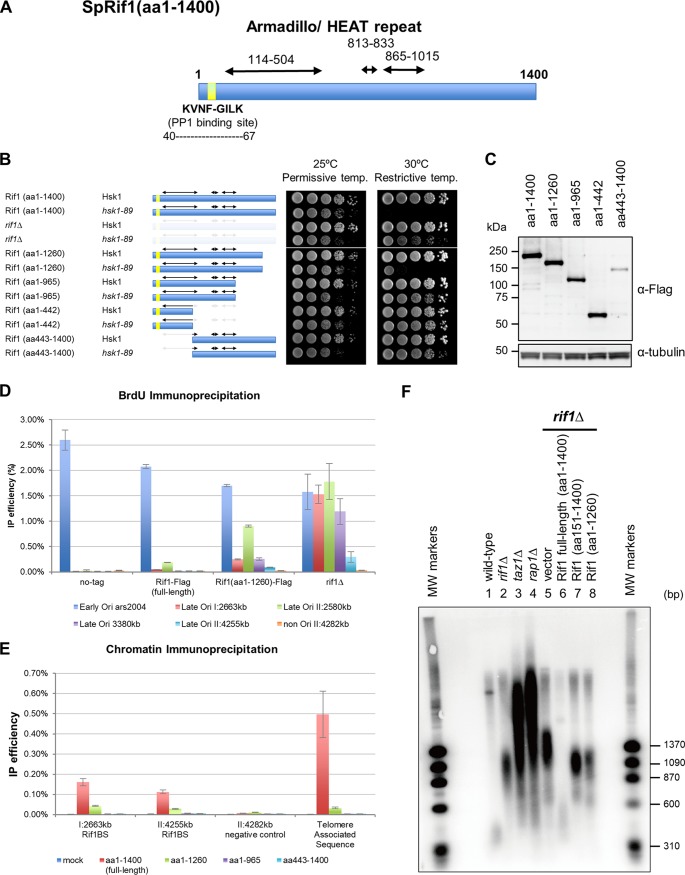
Bypass of the Hsk1 function, origin suppression, and chromatin binding activities of various Rif1 truncation polypeptides. (A) Schematic drawing of fission yeast Rif1 protein (1,400 amino acids). The three conserved armadillo/HEAT repeat segments identified by using the “SUPERFAMILY” search method are indicated by double-arrowed bars. The yellow segment represents conserved PP1 binding sites. (B) Tenfold serial dilutions of exponentially growing cells, as indicated, were spotted onto YES agar and incubated for 5 days at 25 or 30°C. (C, upper) Cell extracts were prepared from exponentially growing Flag-tagged Rif1, Rif1(aa1-1260), Rif1(aa1-965), Rif1(aa1-442), and Rif1(aa443-1400) cells, and the expression levels of truncated Rif1 polypeptides were estimated by Western blotting using anti-Flag antibody. (Lower) Anti-α-tubulin antibody (loading control). (D) Cells expressing the indicated Rif1 truncation polypeptide were arrested at M phase by *nda3-KM311* at 20°C and released into the cell cycle in the presence of 25 mM HU and 200 μg/ml BrdU for 60 min at 30°C. The genomic DNA was extracted, and BrdU-substituted DNA was immunoprecipitated. IP efficiency (relative to the input genomic DNA) was assessed by quantitative real-time PCR. *ars2004* is an early-firing origin, and *Ori* I:2663kb, *Ori* II:2580kb, *Ori* II:3380kb, and *Ori* II:4255kb are late/dormant origins that normally do not fire in the presence of HU. Non-*Ori* II:4282kb is a nonorigin sequence (negative control). Independent experiments were conducted three times. (E) Cells expressing the indicated Rif1 truncation polypeptide were arrested at M phase by *nda3-KM311* mutation and released into the cell cycle at 30°C. The cells were fixed at 15 min after release. Rif1 chromatin immunoprecipitation was conducted, and the chromatin binding of Rif1 was assessed by quantitative real-time PCR at the sites indicated. I:2663kb and II:4255kb are known Rif1 binding sites, and II:4282kb is a negative-control site. Telomere-associated sequence is derived from the sequences located at the end of chromosomes 1 and 2. Independent experiments were conducted three times. (F) Wild-type (YM71), *rif1*Δ (FY14160), *taz1*Δ (FY14161), and *rap1*Δ (FY14171) cells were grown in YES. *rif1*Δ or *rif1*Δ cells expressing full-length or truncated Rif1 were grown in Edinburgh minimal medium without thiamine for 12 h. Cells were collected and genomic DNA was isolated by using a blood and tissue kit (Qiagen). Genomic DNA, digested by ApaI, was separated on 1% agarose gel, and then the telomere was detected by Southern hybridization using a probe containing the TAS1 sequence.

Rif1 is evolutionarily conserved, and the N-terminal segment is composed of many HEAT- and armadillo-type repeats (29), which are helical folds that form extended curved proteins or RNA interface surfaces. The N-terminal segment [Rif1(aa110-471)] is particularly well conserved and regarded as a Rif1 N-terminal domain. Rif1 has a PP1-interacting motif near its N terminus (Fig. 1A) (38). However, other structural and functional domains are not known. We previously reported that disruption of *rif1* can suppress *hsk1*Δ, suggesting that loss of *rif1* can bypass the requirement of Hsk1 function for growth (8). We have generated various truncation forms of Rif1 and examined if they can bypass Hsk1 function. We used an *hsk1-89* mutant which does not grow at 30°C but can grow at 30°C under the *rif1*Δ background. The C-terminal 435-amino-acid truncation [Rif1(aa1-965)] as well as the N-terminal 442-amino-acid truncation [Rif1(aa443-1400)] resulted in efficient suppression of temperature sensitivity of *hsk1-89* (Fig. 1B). Since the efficiency of suppression correlates with the loss of its activity to suppress origin firing, the results indicate both N-terminal and C-terminal segments of Rif1 are important for origin suppression. The truncated polypeptides were expressed at a similar level, except for Rif1(aa443-1400), which is unstable (Fig. 1C). The C-terminal 140-amino-acid truncation (aa 1 to 1260) resulted in partial suppression (Fig. 1B), indicating that the mutant still retains some activity to suppress origins. Consistent with this prediction, bromodeoxyuridine (BrdU) immunoprecipitation in the presence of hydroxyurea (HU) showed that origin firing at late origins was partially deregulated in *rif1*Δ1261-1400 [Rif1(aa1-1260)] (Fig. 1D). The chromatin immunoprecipitation-quantitative PCR (ChIP-qPCR) analyses indicated that Rif1(aa1-965) and Rif1(aa443-1400) completely lost their ability to bind to Rif1BS (Fig. 1E), whereas Rif1(aa1-1260) binds to the Rif1BS on the arm with affinity much lower than that of the full-length protein. The binding to telomere was almost completely lost in all truncated polypeptides (Fig. 1E). Both Rif1(aa151-1400) and Rif1(aa1-1260) failed to correct the telomere deficiency of *rif1*Δ cells, whereas the full-length Rif1 restored the normal telomere (Fig. 1F, lanes 6 to 8). This indicates that both N-terminal and C-terminal segments of Rif1 are required for its chromatin binding, for origin suppression as well as for telomere regulation.

### Both N-terminal and C-terminal polypeptides of Rif1 bind to G4.

**FIG 2:**
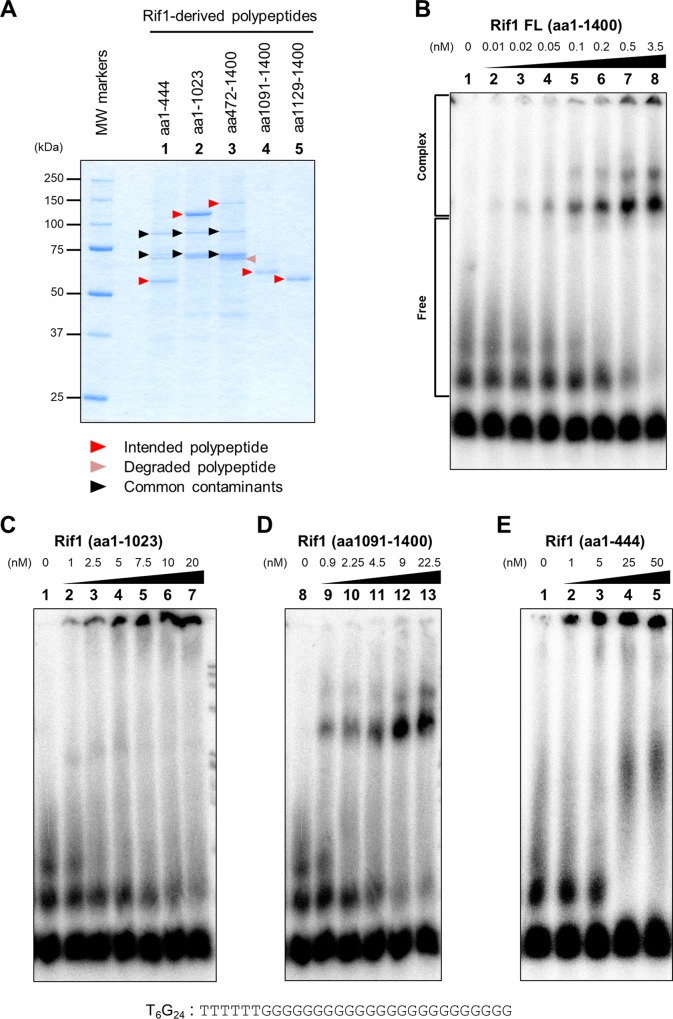
DNA binding activity of truncated polypeptides of Rif1 protein. (A) N-terminally or C-terminally truncated polypeptides of Rif1 protein, expressed in 293T cells and purified or partially purified by an anti-FLAG antibody column, were analyzed by 10% (37.5:1) SDS-PAGE and were detected by Coomassie brilliant blue staining. In the polypeptides containing HEAT repeats (lanes 1 to 3), common contaminants (probably cellular heat shock proteins) were detected. (B to E) ^32^P-labeled T_6_G_24_ oligonucleotide (0.25 pmol; sequence shown below the panels), heat denatured and renatured in 50 mM KCl and 40% PEG 200, was incubated in the presence of increasing amount of the full-length (B), N-terminal (aa 1 to 1023) (C), C-terminal (aa 1091 to 1400) (D), and short N-terminal (aa 1 to 444) (E) polypeptides of Rif1. The samples were analyzed by 8% PAGE (1× TBE, 50 mM KCl, and 40% PEG 200). The related data for this set of experiments where binding constants were estimated are shown in Fig. S1.

We expressed various portions of Rif1, purified them (Fig. 2A), and examined their G4 binding activity. All of the polypeptides containing either an N-terminal or C-terminal segment (aa 1 to 444, aa 1 to 1023, aa 472 to 1400, aa 1091 to 1400, and aa 1129 to 1400) bind to G4 DNA (Fig. 2; see also Fig. S1 in the supplemental material).

Both an N-terminal polypeptide [Rif1(aa1-1023)] and a C-terminal polypeptide [Rif1(aa1091-1400)] bound to G4 with apparent dissociation constants (*K_d_*) of 13.4 and 11.8 nM, respectively (Fig. S1). A shorter N-terminal polypeptide [Rif1(aa1-444)] also bound with a *K_d_* of 15.2 nM (Fig. S1). In contrast, the full-length Rif1 bound to G4 DNA with a *K_d_* of 0.3 to ∼0.8 nM (Fig. S1), roughly consistent with a previous report (45). Thus, both C-terminal and N-terminal polypeptides bind to G4 with affinity lower than that of the full-length polypeptide. We noted that the Rif1(aa472-1400) polypeptide containing both the C-terminal DNA binding domain and a part of the HEAT repeats bound to G4 at a significantly higher level than other N-terminal or C-terminal polypeptides, with an apparent *K_d_* of 0.6 nM (Fig. S1C). Portions of conserved HEAT motifs present on this polypeptide appear to increase the affinity to G4.

DNA binding activity was previously reported for a N-terminal domain of S. cerevisiae Rif1 protein as well (44). Both N-terminal polypeptides [Rif1(aa1-1023) and Rif1(aa1-444)] generate large aggregates at the top of the gel and a small amount of fast-migrating shifted bands in gel shift assays (Fig. 2C and E). The complexes that entered the gel appear to be somewhat smeared, suggesting that they are not very stable during electrophoresis. In contrast, the polypeptides containing the C-terminal segment generate discernible shifted bands with less large aggregates at the top of the gel (Fig. 2D). These observations suggest that the N-terminal and C-terminal polypeptides generate G4-protein complexes with distinct assembly.

**FIG 3:**
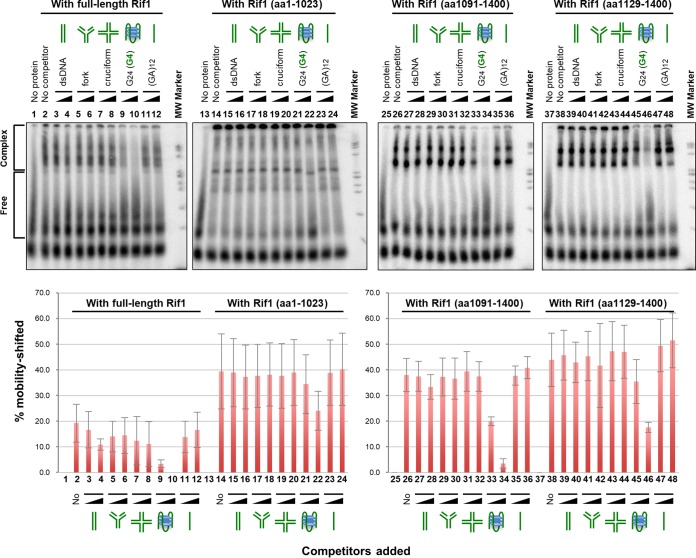
Competition assays with various structured DNAs for G4 binding of truncated polypeptides of Rif1. The labeled T_6_G_24_ DNA (0.2 pmol), heat denatured and renatured in 50 mM KCl and 40% PEG 200, was incubated with full-length Rif1 (0.2 nM) or truncated polypeptides of Rif1 (aa 1 to 1023, 20 nM; aa 1091 to 1400, 10 nM; aa 1129 to 1400, 20 nM) in the presence of various nonlabeled structured DNAs (0.2 and 1 pmol), and samples were analyzed by 10% PAGE (0.5× TBE, 50 mM KCl, and 40% PEG 200). The lower panel shows the quantification of binding, conducted as described in the legend to Fig. S1.

With all the polypeptides, binding was competed most effectively by cold G4 DNA (T_6_G_24_) itself (Fig. 3, lanes 9, 10, 21, 22, 33, 34, 45, and 46; Fig. S2 shows additional details) but not by other structured DNAs, including cruciform, forked DNA, and non-G4 DNA (T_6_[GA]_12_). This was most clearly observed with the C-terminal polypeptides [Rif1(aa1091-1400) and Rif1(aa1129-1400)]. However, competition was weak with the N-terminal polypeptide [Rif1(aa1-1023)] under the same conditions (Fig. 3, lanes 21 and 22). In particular, the high-molecular-weight aggregates at the wells were not significantly reduced by addition of competitor G4 DNA (Fig. 3, lane 22). This could be due to the nature of the N-terminal polypeptides. In a native gel, they mostly run at the top of the gel, indicative of large aggregates (data not shown). In gel shift assays with polyacrylamide gel, the polypeptide aggregates may trap the added competitor DNA at the well. In pulldown assays, both Rif1(aa1-1023) and Rif1(aa1-444) polypeptides exhibited specificity to G4 DNA compared to that of non-G4 DNA (Fig. S3, lanes 4, 5, 9, and 10). The expected N-terminal polypeptides are enriched after pulldown with the G4 oligonucleotide, indicating the absence of other major DNA binders in contaminating polypeptides (Fig. S4, lanes 2 and 7). These results suggest that the dual G4 binding by the C-terminal and N-terminal segments of Rif1 support its G4 recognition/binding, and that the N-terminal HEAT/armadillo repeats facilitate the generation of high-molecular-weight complexes through their self-associating activity.

### Amino acid residues required for G4 binding and oligomerization of Rif1.

**FIG 4:**
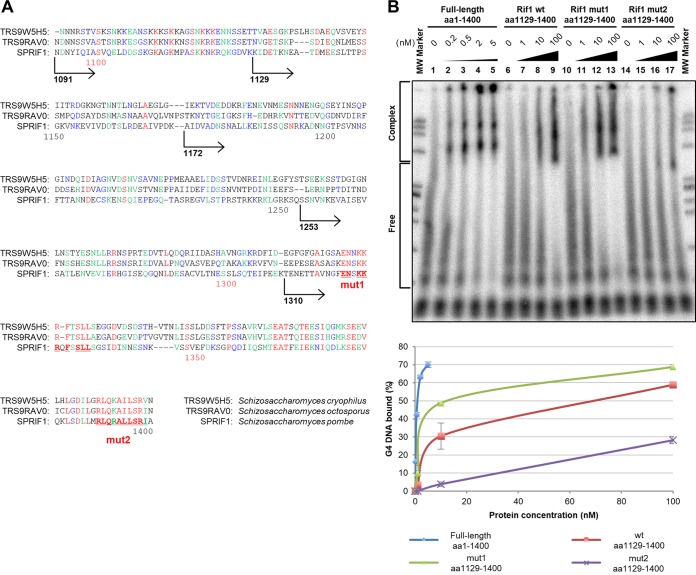
Mutations in the C-terminal conserved residues lead to reduced G4 binding. (A) The C-terminal amino acid sequences of Rif1 protein from three *Schizosaccharomyces* yeasts, S. cryophilus OY26, S. octosporus yFS286, and S. pombe, are aligned. Red, green, and blue letters indicate the identical, conserved, and related amino acid residues, respectively. Numbers below the sequences indicate the amino acid numbers of the S. pombe Rif1. The extents of the four C-terminal polypeptides analyzed in this study are shown, with numbers indicating the residues at their N termini. Two clusters of well-conserved amino acid residues are underlined. These segments were mutated by changing the amino acids shown in boldface letters to alanine (mut1 and mut2). (B) The labeled T_6_G_24_ DNA (0.25 pmol), heat denatured and renatured in 50 mM KCl and 40% PEG 200, was incubated with various amounts of full-length protein (lanes 2 to 5), the wild type (aa 1129 to 1400; lanes 7 to 9), mut1 (aa 1129 to 1400; lanes 11 to 13), or mut2 (aa 1129 to 1400; lanes 15 to 17), and samples were analyzed by 8% PAGE (1× TBE, 50 mM KCl, and 40% PEG 200). The graph shows quantification of the binding, conducted as described in the legend to Fig. S1. Mutations of the conserved sequences near the C terminus of Rif1 (mut2) reduce the G4 binding activity in gel shift assays (estimated K_d_, ∼250 nM). The result presented is a representative from two independent experiments, and the graph represents the average values. Another set of results is shown in Fig. S5.

Alignment of the C-terminal segment of Rif1 revealed the presence of conserved amino acid sequences, including ENSKKRQFSSLL at aa 1321 to 1332 and RLQRAILSR at aa 1390 to 1398 (Fig. 4A). We generated alanine substitutions at the conserved amino acids in the above-described two segments (underlined residues; mut1 and mut2, respectively) on the C-terminal polypeptide (aa 1129 to 1400). The purified mutant proteins were examined for G4 binding. Although mut1 polypeptide showed DNA binding activity identical to or greater than that shown by the wild-type polypeptide, mut2 showed a >10-fold reduction of DNA binding activity in gel shift assays (Fig. 4B, lanes 7 to 9 and 15 to 17; Fig. S5 provides additional data).

**FIG 5:**
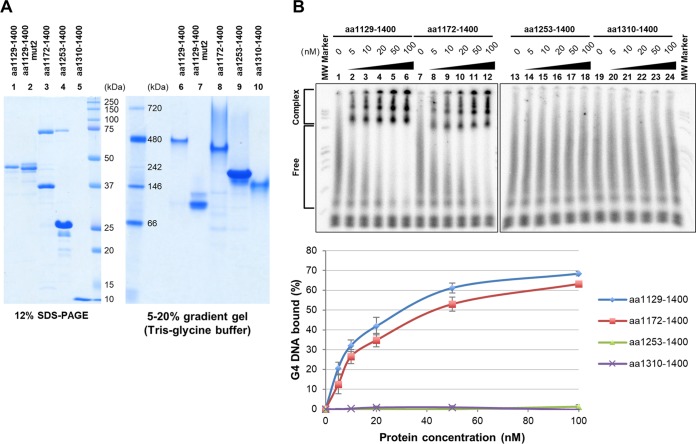
Analyses of multimerization of Rif1 C-terminal polypeptides on a native gel. (A) Rif1 C-terminal polypeptides were analyzed by SDS-PAGE (5% to 20% gradient gel; left, lanes 1 to 5) and on a native protein gel (5% to 20% gradient gel; right, lanes 6 to 10). Proteins were detected by CBB staining. The C-terminal 91 amino acids are sufficient for multimerization, and mutation of the conserved sequences near the C terminus impairs it (mut2, aa 1129 to 1400; lane 7). (B) The labeled T_6_G_24_ DNA (0.25 pmol), heat denatured and renatured in 50 mM KCl and 40% PEG 200, was incubated with various amounts of aa 1129 to 1400 (lanes 2 to 5), aa 1172 to 1400 (lanes 8 to 12), aa 1253 to 1400 (lanes 14 to 18), and aa 1310 to 1400 (lanes 20 to 24), and the samples were analyzed by 8% PAGE (1× TBE, 50 mM KCl, and 40% PEG 200). The bottom graph shows quantification of the binding, conducted as described in the legend to Fig. S1. aa 1172 to 1400 can bind to G4 but not aa 1253 to 1400 or aa 1310 to 1400. In this experiment, the polypeptides derived from C-terminal segments were expressed and purified from E. coli. The result presented is a representative of two independent experiments, and the graph represents the average values. Another set of results is shown in Fig. S6.

Since we previously showed that Rif1 forms oligomers (46; R. Fukatsu, K. Moriyama, and H. Masai, unpublished data), we then examined if the C-terminal polypeptide forms multimers and if the mutation affects the multimerization activity of the protein. We analyzed the purified proteins on a native protein gel. The C-terminal 272-aa (aa 1129 to 1400, 30 kDa [predicted molecular weight]), 229-aa (aa 1172 to 1400, 25 kDa), 148-aa (aa 1253 to 1400, 16 kDa), and 91-aa (aa 1310 to 1400, 10 kDa) polypeptides migrated at ∼480 kDa, ∼400 kDa, ∼240 kDa, and 160 kDa, respectively, on a native gel (Fig. 5A, lanes 6, 8, 9, and 10), suggesting that the C-terminal polypeptides form multimers. The molecular sizes predict the multimers potentially are composed of as many as 16 subunits, although it is difficult to accurately predict the numbers of subunits in each multimer, since the mobility on PAGE is affected by the shape of the molecule as well. The results indicate that the C-terminal 91 amino acids are capable of multimerization, although they do not bind to G4 (Fig. 5B; Fig. S6 provides additional data). Interestingly, the aa 1129 to 1400 mut2 polypeptide migrated at ∼120 kDa (Fig. 5A, lane 7), suggesting that the mutant forms an oligomer with smaller numbers of subunits. In gel filtration, the wild-type aa 1129 to 1400 polypeptide migrated at around 500 to ∼600 kDa, whereas the mut2 aa 1129 to 1400 polypeptide migrated at ∼150 kDa (Fig. S6), consistent with the results of the native gel, showing the importance of the C-terminal conserved sequences for oligomerization. These results indicate that the very C-terminal amino acid residues of Rif1 play a crucial role in G4 DNA binding as well as in oligomerization.

Further analyses of C-terminally truncated Rif1 polypeptides indicated that the C-terminal 229 amino acids (1172 to 1400) are sufficient for G4 binding, whereas the C-terminal 148-amino-acid polypeptide (aa 1253 to 1400) did not bind to DNA (Fig. 5B; Fig. S6 provides additional data), indicating that the 81 amino acids (aa 1172 to 1252) are essential for G4 binding and that the ability to form a large multimer is not sufficient for G4 binding.

### Complexes between Rif1 polypeptides and G4.

The presence of dual DNA binding domains suggests a single molecule of Rif1 binds to more than one molecule of G4. In order to assess more precisely the number of G4 molecules bound by a single molecule of Rif1, we conducted gel shift assays in the presence of excess amounts of the G4 oligonucleotide. The amount of the bound substrate DNA was determined by measuring the radioactivity of the bound (complex) and unbound (free) DNA bands.

**FIG 6:**
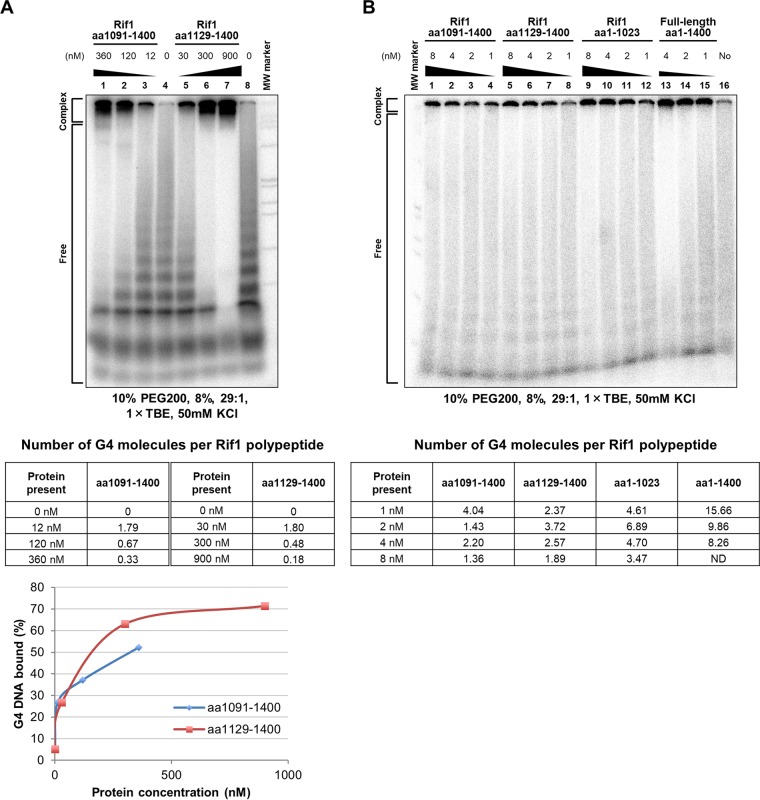
Stoichiometry of G4 molecules bound to Rif1 protein. (A) Gel shift assays were conducted with the Rif1 C-terminal polypeptides (aa 1091 to 1400 and aa 1129 to 1400) as described in the legend to Fig. S1, except that 2.5 pmol of labeled T_6_G_24_ DNA was used as substrate DNA. The samples were analyzed by 8% PAGE (29:1) in 1× TBE, 50 mM KCl, and 10% PEG 200. After an autoradiograph was taken, dried gel pieces containing the free and complex DNA bands were excised from each lane, and the radioactivity was measured in a scintillation counter. The radioactivity in the complex band was divided by the sum of radioactivity of the free and complex DNA, and the amount of template DNA bound with the polypeptide was estimated. On the assumption that all of the polypeptides are involved in the binding, the number of the G4 molecules per molecule of the polypeptide was calculated for each binding (insert tables). (B) Full-length and truncated polypeptides, as shown, were used for gel shift assays, as described for panel A. The number of G4 molecules per molecule of each polypeptide was calculated at different concentrations of peptide, as described for panel A, and the values are presented in the table. Under this condition, >90% of the polypeptides added in the reactions are present in the bound complexes (confirmed by Western blotting).

In order to get better separation of bound and unbound G4, we used a different gel condition (10% polyethylene glycol 200 [PEG 200] instead of 40%). Under this condition, the bound G4 migrates near the well, giving a better separation from the unbound G4. Another feature of this new gel system is that it can separate oligomers of the T_6_G_24_ DNA, known to from a parallel-type G4. ^32^P-labeled T_6_G_24_ DNA (2.5 pmol) was used as a substrate (final concentration of 250 nM). Under this gel condition, the T_6_G_24_ DNA appeared as a clear ladder, indicative of the presence of dimer, trimer, tetramer, etc. In titration of the C-terminal polypeptides (aa 1091 to 1400 and aa 1129 to 1400), the larger T_6_G_24_ DNAs were bound at the lowest concentration of the polypeptide, and the smaller G4 elements were bound only at a higher concentration (Fig. 6A), as was predicted from the results for other gels (Fig. 2). These results indicate the preference of the Rif1 C-terminal G4 binding domain for the polymers of G4.

A molecule of the above-described C-terminal polypeptides bound close to two molecules of the G4 DNA, on average, at a low protein concentration (12 nM). This is probably due to its preferential binding to the oligomers of G4. At a high concentration (∼300 nM; almost equimolecular to the G4 DNA), the number of G4 molecules per molecule of C-terminal polypeptide dropped to 0.3 to 0.5. The results suggest that the stoichiometry of G4 per monomer of the C-terminal polypeptide is low; the polypeptide likely binds to a single molecule of G4. In contrast, the preparation containing the full-length polypeptide bound to the G4 substrate with stoichiometry of >15 G4 molecules at 1 nM protein and >8 G4 molecules per molecule of the polypeptide even at 4 nM protein (Fig. 6B). Interestingly, the N-terminal polypeptide (aa 1 to 1023) also showed stoichiometry of ∼7 G4 (at 2 nM) and >3 G4 (at 8 nM) molecules. In contrast, the number of G4 molecules per molecule of the C-terminal polypeptide did not exceed 4 even at 1 nM and was less than 2 at 8 nM (Fig. 6B). By Western blot analyses of the same binding samples, we have confirmed that >90% of the polypeptides added are involved in binding to G4 at a concentration of 8 nM (data not shown). These results suggest a role of the HEAT domain in coordinating the binding of Rif1 to multiple G4 molecules in conjunction with the C-terminal G4 binding domain rather than the possibility that the two G4 binding domains on Rif1 independently bind to G4.

### Identification of point mutant Rif1 capable of bypassing Hsk1 function.

**FIG 7:**
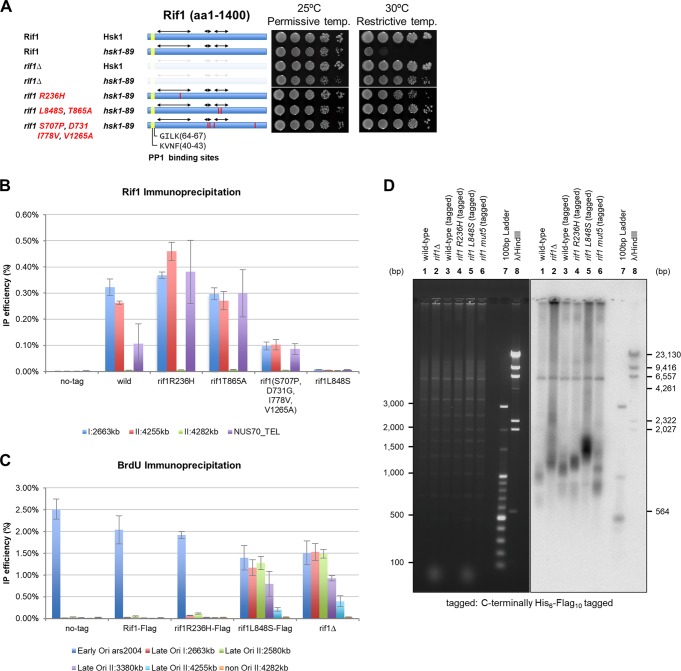
Isolation of *rif1* mutants that can bypass Hsk1 function and its chromatin binding and origin suppression activities. (A) Tenfold serial dilutions of exponentially growing cells, as indicated, were spotted onto YES agar and incubated for 5 days at 25 or 30°C. Red bars indicate the positions of amino acid substitutions. (B) Cells carrying the indicated *rif1* mutation (tagged with His_6_-Flag_10_ at the C terminus) were arrested at M phase by *nda3-KM311* at 20°C and released into the cell cycle at 30°C. The cells were fixed 15 min after release. Rif1 chromatin immunoprecipitation was conducted, and the chromatin binding of Rif1 was assessed by quantitative real-time PCR at the sites indicated. I:2663kb and II:4255kb are known Rif1 binding sites, and II:4282kb is a negative-control site. NUS70_TEL is located at a telomere. (C) Cells carrying the indicated *rif1* mutation were arrested at M phase by *nda3-KM311* at 20°C and released into the cell cycle in the presence of 25 mM HU and 200 μg/ml BrdU for 60 min at 30°C. The genomic DNA was extracted, and BrdU-substituted DNA was immunoprecipitated. The IP efficiency (relative to the input genomic DNA) was assessed by quantitative real-time PCR. The origins examined are the same as those described for Fig. 1D. In panels B and C, independent experiments were conducted three times. (D) The indicated cells were collected and genomic DNA was isolated by using a blood and tissue kit (Qiagen). Genomic DNA, digested by EcoRI, was separated on 1% agarose gel, and then telomere was detected by Southern hybridization using a probe containing the TAS1 sequence.

In order to identify novel motifs involved in Rif1’s origin suppression function, we screened mutants of Rif1 which could rescue the growth of *hsk1-89* at 30°C (Fig. S8 depicts the strategy). Six mutants were identified, and mutation sites were determined (Fig. S9). Generally, the mutants contain multiple amino acid substitutions, and we tried to identify the responsible mutations by constructing mutants harboring each single-amino-acid substitution. We have identified R236H and L848S, each of which alone could suppress *hsk1-89*. L848S suppressed *hsk1-89* to an extent similar to that by *rif1*Δ, while R236H did so slightly weakly (Fig. 7A and Fig. S9). The single L848S mutation suppressed *hsk1-89* as efficiently as the L848S T865A double mutant, whereas T865A, which coexisted with L848S in mut4, did not restore the growth of *hsk1-89* at 30°C (data not shown); thus, the L848S T865A double mutant can be regarded as being identical to the L848S single mutant. We next examined the chromatin binding of these mutant Rif1 proteins at two Rif1BS (Rif1BS_I:2663_ and Rif1BS_II:4255_) and a telomere (NUS70_TEL). R236H bound to both Rif1BS and to the telomere as efficiently as or more efficiently than the wild type. On the other hand, L848S did not bind to either of the two Rif1BS or to the telomere. T865A bound to chromatin as efficiently as the wild type, consistent with its no-effect phenotype (Fig. 7B). mut5 (S707P D731G I778V V1265A) bound to the two Rif1BS less efficiently but bound to telomere as efficiently as the wild type (Fig. 7B). BrdU incorporation was deregulated in the L848S mutant at four late-firing origins, Ori_I:2663_, Ori_II:2580_, Ori_II:3380_ (*ars727*), and Ori_II:4255_, as vigorously as in *rif1*Δ (Fig. 7C). In the R236H mutant, DNA synthesis at Ori_I:2663_ and Ori_II:2580_, but not at Ori_II:3380_ or Ori_II:4255_, was weakly deregulated (Fig. 7C). The elongated telomere in *rif1*Δ was corrected by the wild-type as well as by the R236H mutant Rif1 protein. However, the L848S mutant could not restore normal telomere. The mut5 mutant partially restored telomere length (Fig. 7D). Thus, the L848S mutant, incapable of chromatin binding, has lost the functions for both origin suppression and telomere regulation, whereas R236H, capable of binding to chromatin, appears to be defective more specifically in origin regulation at some selected late-firing origins. The results establish that chromatin binding of Rif1 is essential for its capability for origin firing, and they indicate a role of additional interfaces (in addition to N- and C-terminal domains) on Rif1 protein in its chromatin binding and that not only chromatin binding but also other unknown functions of Rif1 are required for its origin suppression activity.

## DISCUSSION

Rif1 is a conserved nuclear protein that was initially identified as a telomere binding factor in yeasts (14, 15). In higher eukaryotes, in contrast, Rif1’s role in telomere regulation is diminished, but it plays important roles in pathway choice of repair of double-stranded DNA breaks (30–34). Rif1 stimulates NHEJ repair through association with 53BP1. We and others reported crucial roles of Rif1 in replication timing regulation that are conserved from yeasts to human (8–10). Although its DNA binding activity and PP1 binding sites have been characterized (29, 38–43, 46–49), how these and other domains of Rif1 are coordinated in its function of spatiotemporal regulation of DNA replication remains unsolved.

### Fission yeast Rif1 C-terminal segment contains both G4 DNA binding and multimerization activities.

Rif1 binding sites (Rif1BSs) on the chromosome, as determined by ChIP sequencing (ChIP-seq), contains conserved sequence motifs (Rif1CSs) composed of 5 to 6 G tracts. Mutagenesis analyses indicated functional significance of these G tracts for Rif1 binding and for G4 formation *in vitro* (45). Purified Rif1 binds selectively to G4 *in vitro*, suggesting direct recognition of G4 by Rif1. Gel filtration and glycerol gradient analyses indicate that Rif1 forms tetramers, octamers, or even larger oligomers (our unpublished data).

Analyses of polypeptides derived from the C-terminal segments on a native gel clearly indicated the formation of a distinct oligomer (Fig. 5A), which was estimated to be a multimer containing 16 subunits at maximum. The C-terminal 91 amino acids were sufficient for generation of a multimer. We have mapped the G4 DNA binding domain within the C-terminal 229-amino-acid polypeptide, and a further 81-amino-acid deletion led to loss of DNA binding. Furthermore, amino acid substitutions of the conserved sequences near the C terminus (mut2) resulted in reduced DNA binding and partial destabilization of oligomer formation. mut2 generated an oligomer of reduced size, probably a tetramer or dimer. The analyses on gel filtration also supported this conclusion (see Fig. S7 in the supplemental material). Thus, the C-terminal conserved sequences are required for both high-affinity G4 binding and stable formation of a large oligomer (Fig. 4 and 5), suggesting that G4 binding is functionally linked to multimer formation. Since the shapes of the polypeptides affect migration on a native gel or in gel filtration, we cannot rule out the possibility that these C-terminal polypeptides are tetramers or octamers (with mut2 aa 1129 to 1400 being a monomer or a dimer).

It has been reported that the C-terminal segments of Rif1 from budding yeast and mammalian cells contain DNA binding activity and can oligomerize. aa 2170 to 2246 of human Rif1 was reported to bind to fork and Holliday junction (HJ) DNA (29). The C-terminal 60 aa (CTD) from budding yeast Rif1 protein forms a tetramer and binds to Rap1 (44). More recently, CRII (aa 2226 to 2340) from mouse Rif1 was reported to constitute a structure-specific DNA binding domain (48). Although this domain has similarity to the tetramerization domain of budding yeast Rif1, it did not show clear oligomerization activity.

### The N-terminal segment of Rif1 also binds to DNA in a G4-specific manner.

We discovered that the N-terminal HEAT motif segments also contain DNA binding activity (Fig. 2 and Fig. S1). In gel shift assays on G4, the N-terminal polypeptides tend to generate high-molecular-weight complexes (trapped at the top of the gel) as well as a small amount of lower-molecular-weight complexes that appear as broad bands (Fig. 2). Indeed, the majority of the N-terminal polypeptides migrate as high-molecular-weight complexes (aggregates at the well) on a native gel (data not shown). In pulldown assays, the N-terminal HEAT motif polypeptides also showed specific binding to G4 (Fig. S3). Recently, structures of the N-terminal domain of S. cerevisiae Rif1 (ScRif1-NTD) were solved (47). The ScRif1-NTD adopts a unique shepherd’s crook shape, the hook of which is involved in capturing DNA. ScRif1-NTD forms a dimer and was proposed to spread on DNA. Similar mechanisms may operate on high-molecular-weight complex formation by fission yeast Rif1 N-terminal polypeptides on G4. ScRif1-NTD was shown to be essential for telomere length regulation and for the telomeric anticheckpoint function (47).

In contrast to the N-terminal polypeptides, the C-terminal polypeptides generate distinct shifted bands on G4. These results reveal aspects on the molecular nature of the N-terminal and C-terminal polypeptides, which may interact with DNA in distinct manners. The affinity of the full-length Rif1 to G4 is at least severalfold higher than that of the C-terminal or N-terminal fragment (Fig. S1), suggesting that the N-terminal HEAT and C-terminal G4 binding domains (delineated to 444 and 229 aa, respectively) are coordinately involved in high-affinity and stable binding of Rif1 to DNA. The dual G4 binding domain structure is also shared by mammalian Rif1 protein (46).

### Dissection of functional domains of Rif1 protein in regulation of replication timing.

**FIG 8:**
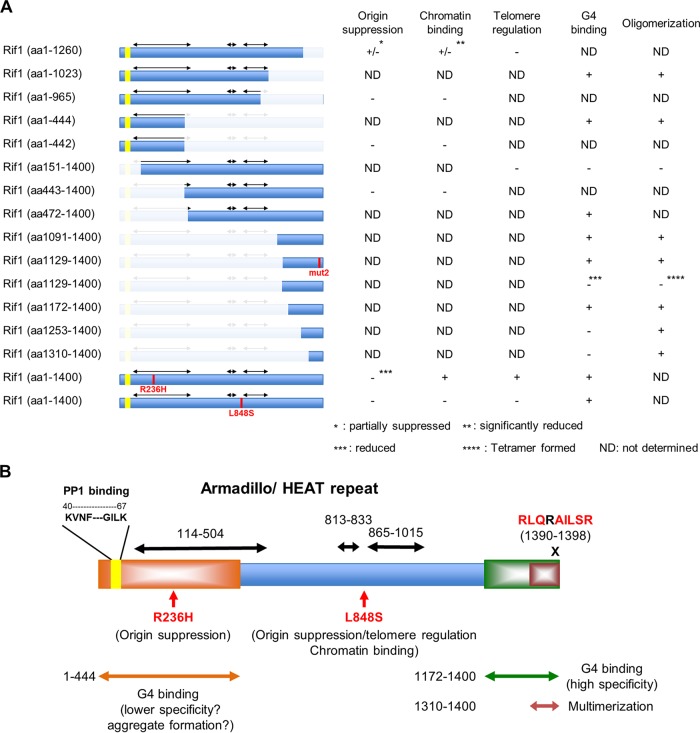
Summary of the results. (A) Summary of the properties of the mutant Rif1 proteins constructed and analyzed in this study. Chromatin binding represents binding to Rif1BS on the arm as determined by ChIP-qPCR/ChIP-Seq. G4 binding represents *in vitro* binding of the purified protein to the G4 DNA. (B) Schematic drawing of the functional segments of fission yeast Rif1 protein determined in this study. Among the three conserved armadillo/HEAT repeat segments (shown by double-arrowed bars) identified by using the “SUPERFAMILY” search method, the longest armadillo/HEAT repeat (aa 114 to 514) largely overlaps the Rif1 core sequence, the segment showing the highest conservation from yeast to human. Two novel mutations of *rif1* that impair origin suppression activity are shown. The conserved RLQRAILSR (aa 1390 to 1398) sequences, important for G4 binding and multimerization, are indicated. Deletion of either N-terminal (Δ1-442; aa 443 to 1400) or C-terminal (Δ966-1400; aa 1 to 965) sequence led to complete loss of origin suppression activity. Deletion of the C-terminal 140 amino acids (aa 1 to 1260) also led to partial but significant loss of origin suppression and chromatin binding.

In spite of aberrant spatiotemporal regulation of DNA replication, fission yeast *rif1*Δ cells are quite healthy and grow normally. They do not show sensitivity to replication stress or to DNA damage agents (data not shown). We have shown that the ability of *rif1*Δ to bypass Hsk1 (or to suppress temperature sensitivity of *hsk1-89*) reflects the loss of origin suppression activity; therefore, we utilized this characteristic of *rif1*Δ to measure the origin suppression activity of each mutant (Fig. 8).

Analyses of truncation mutants indicate that deletion of either the N-terminal 442 or C-terminal 435 amino acids results in complete suppression of *hsk1-89*, i.e., loss of origin suppression (Fig. 1B). Neither truncation mutant binds to Rif1BS *in vivo* (Fig. 1E), supporting the roles of both N-terminal and C-terminal segments in chromatin binding. The deletion of the C-terminal 140 amino acids, which is a part of the C-terminal G4 binding domain, led to partial suppression of *hsk1-89* and caused firing at late-firing origins at the level 20% to 50% of that observed in *rif1*Δ. The mutant also exhibited significantly reduced chromatin binding at Rif1BS (20% to 25% at the Rif1BS arm and less than 10% at telomeres) (Fig. 1E). The N-terminal 442 amino acids contain PP1 binding sites (KVNF-GILK; present at aa 40 to 67), known to be essential for origin suppression; thus, loss of origin suppression in Rif1(aa443-1400) is likely to be caused by its inability to recruit PP1. However, R236H mutation, located in the conserved HEAT repeat and not expected to affect PP1 recruitment, impairs origin suppression, supporting the role of the N-terminal HEAT repeat in origin suppression. The HEAT repeats, which are required for telomere and Rif1BS arm binding, as shown above, are required for localization of Rif1 at DSB in mammalian cells as well (32). Thus, diverse cellular roles of Rif1 depend on the conserved N-terminal HEAT repeats. Results described above indicate the functional significance of G4 binding mediated by both N-terminal (HEAT domain delineated to 444 aa) and C-terminal (delineated to 229 aa) segments of Rif1 in its origin suppression activity. The combined actions of the two domains may generate a nucleoprotein complex required for replication timing regulation.

### How does Rif1 contribute to the formation of chromatin architecture that is suppressive for origin activation?

**FIG 9:**
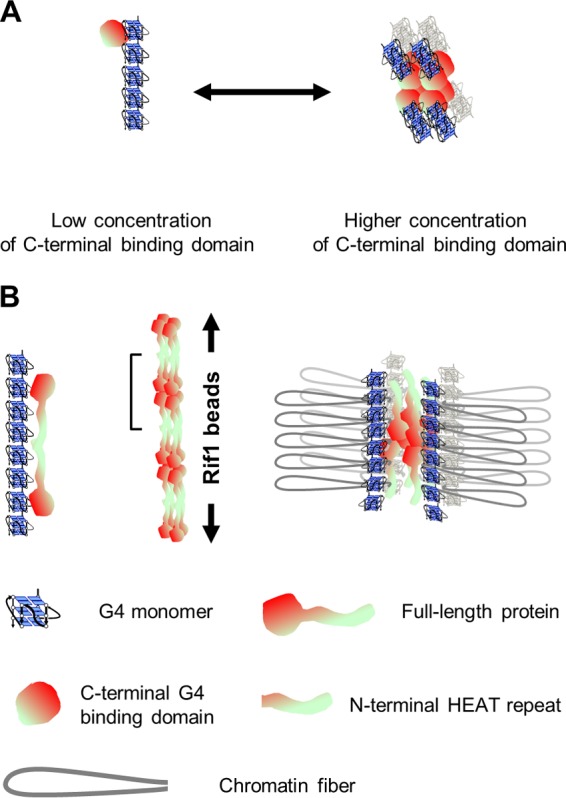
Model of how Rif1 protein organizes chromatin fibers. (A) A single C-terminal G4 binding domain polypeptide binds to a single molecule of G4. (Left) At lower concentration, it selectively binds to multimerized G4, resulting in more than one molecule of G4 bound to a single C-terminal polypeptide. Only a monomer of an oligomeric Rif1 C-terminal polypeptide is depicted. (Right) Each monomer of an oligomeric C-terminal polypeptide (depicted as an octamer in this figure) can bind to a single G4 molecule. At a higher concentration of the C-terminal polypeptide *in vitro*, G4 binds to low-molecular-weight G4s (such as dimers or trimers), resulting in fewer than one molecule of G4 per molecule of the C-terminal polypeptide on average. (B) The presence of the N-terminal HEAT motif increases the affinity to G4 and the stoichiometry of interacting G4 molecules per Rif1 molecule. (Left) In the presence of the N-terminal HEAT repeats, Rif1 may make additional interactions through the N-terminal domain, causing a single molecule of Rif1 to interact with multiple G4 molecules. Rif1 is drawn as a dimer for simplicity. (Center) Oligomer formation (octamer) of the C-terminal G4 binding domain generates the Rif1 bead structure. The portion indicated by a bracket represents a putative octameric Rif1 molecule. (Right) Oligomerized Rif1 (octamer) may interact with multiple chromatin fibers through G4 binding, generating chromatin loops that may constitute replication-inhibitory chromatin domains. Here, we speculate that G4 generated in the intergenic segments on the genome interact with each other, generating multimerized G4, which contributes to the formation of chromatin loops.

Quantitative analyses of Rif1 binding to G4 suggest a single molecule of the C-terminal G4 binding polypeptide binds to a single molecule of G4 (Fig. 6). In contrast, a single molecule of the full-length Rif1 protein appears to interact with multiple molecules of G4 DNA. Furthermore, the N-terminal polypeptide containing HEAT repeats (aa 1 to 1023) is capable of binding to multiple molecules of G4, albeit with reduced stoichiometry compared to that of the full-length version. We speculate that each subunit of the full-length Rif1 protein interacts with multimerized G4 through its C-terminal G4 binding domain and the shepherd’s crook-shaped N-terminal HEAT repeats, which may be interacting with those from the adjacent assembly of Rif1. The combined action of the two domains would lead to a stable complex between Rif1 and G4 multimers. Rif1 forms a multimer through its C-terminal domain, and each subunit would bind to the G4 multimers. The target G4s are derived from the Rif1BS on the genome, which we speculate would interact with each other to form G4 multimers. The chromatin fibers between the Rif1BS would be looped out to generate specific chromatin architecture that would be essential for proper replication timing regulation (Fig. 9).

### Point mutants of Rif1 capable of bypassing Hsk1 suggest the presence of additional interfaces required for its origin suppression activity.

We took advantage of the abovementioned *rif1*Δ genotype to isolate novel mutants. Selection of viable *hsk1-89* cells at 30°C after mutagenesis of the *rif1* gene led to identification of 6 mutants that can suppress the *hsk1* mutation (Fig. S8). Mutants generally contained multiple amino acid changes. After separating each mutation, we came up with two mutations, L848S and R236H, each of which is responsible for Hsk1 bypass (Fig. 7). L848S does not bind to Rif1BS or telomere *in vivo*, whereas R236H retains full binding activity (Fig. 7B). L848S also is defective in telomere regulation, but R236H is mostly proficient in telomere regulation (Fig. 7D). PP1 recruitment may not be affected in either mutant, since PP1 binding sites are intact.

The L848S mutant lost origin suppression and telomere regulation activities, probably due to its inability to bind to chromatin. It is not clear how L848S mutation affects chromatin binding of Rif1. The purified L848S protein binds to G4 as efficiently and selectively as the wild-type protein *in vitro* (data not shown), suggesting that G4 binding alone is not sufficient for chromatin binding of Rif1 *in vivo*. Stable binding of Rif1 to chromatin requires not only G4 binding but also some other unknown factors that may be affected by the L848S mutation. The R236H mutation does not affect the *in vivo* chromatin binding but is partially defective in origin suppression. *In vitro*, the R236H mutation does not affect DNA binding activity of the full-length or of the N-terminal polypeptide (data not shown). Thus, chromatin binding is not sufficient for origin suppression, and some other factors that interact with R236 would be required. Alternatively, a part of the HEAT motif structure may be partially disrupted by the mutation, which is located in the middle of the most conserved sequences. Both L848 and R236H are within the segments that are conserved in fission yeast species (data not shown); thus, they may constitute unknown functional interfaces required for the functions of Rif1. Further biochemical and genetic analyses of the mutants would be required to clarify how Rif1 binds to chromatin and regulates chromatin architecture.

## MATERIALS AND METHODS

### Medium for Schizosaccharomyces pombe.

YES medium, containing 0.5% yeast extract, 3% glucose, and 0.1 mg/ml each of adenine, uracil, leucine, lysine, and histidine, was used for cell culture, and YES plates were made by adding 2% agar to YES medium. G418 (0.2 mg/ml) was added to YES medium for selection of KanMX. For 5-fluoroorotic acid (5-FOA) selection, 0.1 mg/ml 5-FOA was added to medium containing 6.3 g/liter synthetic dextrose minimal medium (SD), 2% glucose, and 0.1 mg/ml each of adenine, uracil, and leucine. Yeast strains used in this study are listed in Table 1.

**TABLE 1:** Fission yeast strains used in this study^*a*^

Strain	Genotype	Source
YM71	*h^–^ leu1-32 ura4-D18*	Our stock
KO147	*h^–^ leu1-32 ura4-D18 hsk1-89*:*ura4*^+^	Our stock
FY14160	*h^–^ leu1-32 ura4-D18 rif1*Δ::*ura4*^+^	NBRP^*b*^
KYP1316	*h^–^ leu1-32 ura4-D18 hsk1-89*–Flag_3_:*kanR rif1*Δ::*ura4*^+^	This study
KYP1261	*h^–^ nda3-KM311 leu1-32 ura4-D18* AUR1:*aur1r*-Adh1-TK-Adh1-ENT1 Rif1-His_6_-Flag_10_:*kanR*	Our stock
KYP1262	*h^–^ nda3-KM311 leu1-32 ura4-D18* AUR1:*aur1r*-Adh1-TK-Adh1-ENT1 *rif1*(*aa1-1260*)-His_6_-Flag_10_:*kanR*	This study
KYP1263	*h^–^ nda3-KM311 leu1-32 ura4-D18* AUR1:*aur1r*-Adh1-TK-Adh1-ENT1 *rif1*(*aa1-965*)-His_6_-Flag_10_:*kanR*	This study
KYP1264	*h^–^ nda3-KM311 leu1-32 ura4-D18* AUR1:*aur1r*-Adh1-TK-Adh1-ENT1 *rif1*(*aa1-442*)-His_6_-Flag_10_:*kanR*	This study
KYP1267	*h^–^ nda3-KM311 leu1-32 ura4-D18* AUR1:*aur1r*-Adh1-TK-Adh1-ENT1 *rif1*(*aa443-1400*)-His_6_-Flag_10_:*kanR*	This study
KYP1311	*h^–^ nda3-KM311 leu1-32 ura4-D18* AUR1:*aur1r*-Adh1-TK-Adh1-ENT1 Rif1-His_6_-Flag_10_:*kanR hsk1-89*:*ura4*^+^	This study
KYP1312	*h^–^ nda3-KM311 leu1-32 ura4-D18* AUR1:*aur1r*-Adh1-TK-Adh1-ENT1 *rif1*(*aa1-1260*)-His_6_-Flag_10_:*kanR hsk1-89*:*ura4*^+^	This study
KYP1313	*h^–^ nda3-KM311 leu1-32 ura4-D18* AUR1:*aur1r*-Adh1-TK-Adh1-ENT1 *rif1*(*aa1-965*)-His_6_-Flag_10_:*kanR hsk1-89*:*ura4*^+^	This study
KYP1314	*h^–^ nda3-KM311 leu1-32 ura4-D18* AUR1:*aur1r*-Adh1-TK-Adh1-ENT1 *rif1*(*aa1-442*)-His_6_-Flag_10_:*kanR hsk1-89*:*ura4*^+^	This study
KYP1315	*h^–^ nda3-KM311 leu1-32 ura4-D18* AUR1:*aur1r*-Adh1-TK-Adh1-ENT1 *rif1*(*aa443-1400*)-His_6_-Flag_10_:*kanR hsk1-89*:*ura4*^+^	Our stock
KYP1328	*h*^+^ *nda3-KM311 leu1-32 ura4-D18* AUR1:*aur1r*-Adh1-TK-Adh1-ENT1 *rif1*(*aa21-1400Δ*)::*ura4*^+^	This study
KYP1601	*h^–^ nda3-KM311 leu1-32 ura4-D18 rif1*(*T557A, S1024N, S1063P*)-His_6_-Flag_10_:*kanR* AUR1:*aur1r*-Adh1-TK-Adh1-ENT1	This study
KYP1602	*h*^+^ *nda3-KM311 leu1-32 ura4-D18 rif1*(*Q16R, A352T, S1361P*)-His_6_-Flag_10_:*kanR* AUR1:*aur1r*-Adh1-TK-Adh1-ENT1	This study
KYP1603	*h^–^ nda3-KM311 leu1-32 ura4-D18 rif1*(*R236H*)-His_6_-Flag_10_:*kanR* AUR1:*aur1r*-Adh1-TK-Adh1-ENT1	This study
KYP1604	*h*^+^ *nda3-KM311 leu1-32 ura4-D18 rif1*(*L848S, T865A*)-His_6_-Flag_10_:*kanR* AUR1:*aur1r*-Adh1-TK-Adh1-ENT1	This study
KYP1605	*h^–^ nda3-KM311 leu1-32 ura4-D18 rif1*(*S707P, D731G, I778V, V1265A*)-His_6_-Flag_10_:*kanR* AUR1:*aur1r*-Adh1-TK-Adh1-ENT1	This study
KYP1607	*h*^+^ *nda3-KM311 leu1-32 ura4-D18 rif1*(*E9G, K635R, S1202G*)-His_6_-Flag_10_:*kanR* AUR1:*aur1r*-Adh1-TK-Adh1-ENT1	This study
KYP1608	*h*^+^ *nda3-KM311 leu1-32 ura4-D18 rif1L848S*-His_6_-Flag_10_ AUR1:*aur1r*-Adh1-TK-Adh1-ENT1	This study
KYP1609	*h*^+^ *nda3-KM311 leu1-32 ura4-D18 rif1T865A*-His_6_-Flag_10_ AUR1:*aur1r*-Adh1-TK-Adh1-ENT1	This study
KYP1610	*h*^+^ *nda3-KM311 leu1-32 ura4-D18 rif1R236H*-His_6_-Flag_10_ AUR1:*aur1r*-Adh1-TK-Adh1-ENT1	This study

a Double colons are used to indicate that the gene of interest is disrupted by insertion of the marker appearing after the double colon. Single colons are used to indicate that the gene of interest is linked to the marker appearing after the single colon.

b NBRP, National Bioresource Project.

### Antibodies.

The antibodies used were M2 (F1804; Sigma-Aldrich) and anti-DDDDK tag monoclonal antibody (FLA-1; MBL). Anti-fission yeast Rif1 antibody was previously reported (45).

### Genetic manipulations for construction of truncation mutants of Rif1.

First, a plasmid containing 5′UTR-Rif1-His_6_-Flag_10_-kanMX-3′UTR sequences was constructed. With the inverse PCR on this plasmid and In-Fusion reaction (TaKaRa Bio Inc.), plasmids carrying 5′UTR-Rif1 (with a truncation)-His_6_-Flag_10_-kanMX-3′UTR sequences were generated. DNA fragments containing 5′UTR-Rif1 (with or without a truncation)-His_6_-Flag_10_-kanMX-3′UTR sequences were used to generate tagged wild-type *rif1* or *rif1* truncation mutants at the endogenous *rif1* locus of the wild-type or *hsk1-89* cells (see Fig. S8 in the supplemental material).

### Strategy for isolation of *rif1* mutants that bypass Hsk1 function.

Deletion of Rif1(Δaa21-1400) in the *hsk1-89* background was generated by integration of the *ura4*^+^ gene into the Rif1 coding frame. Random mutagenesis was conducted on the Rif1 gene by PCR-based mutagenesis. The Rif1 gene segment containing both 5′ and 3′ untranslated regions (UTR) was amplified by PCR with Ex Taq polymerase (TaKaRa Bio) and a primer set (5′-GGATGTTCGTATCGTATATACTG-3′ and 5′-TACCACACAACATCGCAAGCT-3′) in the presence of 80 μM manganese. The constructed mutation library of Rif1 was used to replace the *rif1*(Δ21-1400aa)::*ura4*^+^ site. The cells with mutated *rif1* were isolated by selection at 30°C, the restrictive temperature of *hsk1-89*, in the presence of 5-FOA. Expression of the full-length Rif1 in each candidate clone was confirmed by Western blotting with anti-fission yeast Rif1 antibody. The genomic DNA was isolated from the candidate mutant strains expressing full-length Rif1, and mutations were identified by direct sequencing of the entire Rif1 coding frame (Fig. S8).

### ChIP-qPCR.

Synchronization and ChIP were conducted as previously described (45).

### Expression and purification of fission yeast Rif1 protein and its derivatives.

N-terminally His_6_- and C-terminally Flag_3_-tagged Rif1 protein or its derivatives were cloned into ver3-4 vector, expressed in 293T cells, and purified as previously described (50). The biological functions of Rif1 are not impaired by the addition of the two tags (data not shown). C-terminal polypeptides were expressed in Escherichia coli as well. The coding frame was cloned into pT7-7/QE30 (in which RGS-His_6_ tag sequence from the pQE-30 vector was inserted into the pT7-7 vector containing T7 promoter [51]) to generate N-terminal RGS-His_6_ fusion polypeptides. The proteins were purified with a nickel column and further purified with a Mono Q column, if necessary.

### G4 DNA and Rif1BS DNA.

The G4 oligonucleotide used in the assays was T_6_G_24_ and the control non-G4 oligonucleotide was T_6_(GA)_12_; they were oligonucleotide purification cartridge (OPC) column purified and further purified by PAGE containing 8 M urea before use. Oligonucleotides and duplex DNA were heat denatured at 98°C for 3 min and gradually cooled down to room temperature in 50 mM KCl and 40% PEG 200 (52, 53). In some experiments, oligonucleotides were heat denatured and cooled down in 60 mM cacodylate buffer with 60 mM KCl.

### Analyses of DNA and proteins on polyacrylamide gels.

DNA, heat denatured in formamide containing 5 mM EDTA, was analyzed on PAGE containing 8 M urea. Regular DNA was analyzed in PAGE containing 10% glycerol in 0.5× Tris-borate-EDTA (TBE).

### Gel shift assays.

Labeled DNA fragments or cold DNA fragments were mixed with purified proteins in reaction mixtures (10 μl or 20 μl) containing 40 mM HEPES-KOH (pH 7.6), 50 mM KCl, 1 mM EDTA, 10% glycerol, and 0.01% Triton X-100 with 0.25 pmol of ^32^P-labeled and heat-denatured T_6_G_24_ DNA. In some assays, 2.5 pmol of the same G4 DNA was used in the 10-µl assay mixtures. After incubation at room temperature for 30 min, the reaction mixtures were directly applied onto a polyacrylamide gel prepared in 1× TBE, 50 mM KCl, and 40% PEG 200 or on those prepared in 1× TBE, 50 mM KCl, and 10% PEG 200 in 1× TBE plus 50 mM KCl. In competition assays, labeled DNA and competitor DNA, which were separately heat denatured and reannealed, were premixed, and proteins were added last. The sequences of competitor DNAs are from reference 48 and also are described in Table S3 of reference 45. All of the DNA binding assays were conducted twice or more with similar results, and representative data are shown in the main figures (additional sets of the data for gel shift assays presented in main figures are shown in Fig. S1, S2, S5, and S6).

### Pulldown assays.

Ten picomoles of biotinylated T_6_G_24_ or T_6_(GA)_12_ oligonucleotide and purified SpRif1 derivative polypeptides, as indicated, was mixed in 200 μl of 40 mM HEPES-KOH (pH 7.6), 50 mM KCl, 1 mM EDTA, 10% glycerol, 1 mM dithiothreitol, and 0.01% Triton X-100 and incubated at room temperature for 30 min. After addition of 10 μl of streptavidin Dynabeads (M-280), equilibrated in the buffer described above, biotinylated oligonucleotides were pulled down, washed with 500 μl of binding buffer three times, resuspended in 20 μl of 1 × SDS-PAGE sample buffer, boiled at 96°C for 1 min, and analyzed by 5% to 20% gradient SDS-PAGE, followed by Western analyses with anti-Flag antibody.

## Supplementary Material

Supplemental file 1
