# Supplementary material for: Both a Unique Motif at the C Terminus and an N-Terminal HEAT Repeat Contribute to G-Quadruplex Binding and Origin Regulation by the Rif1 Protein
Source: Mol Cell Biol. 2019 Feb 4;39(4):e00364-18. doi: 10.1128/MCB.00364-18 (PMC6362314; doi:10.1128/MCB.00364-18)
Supplement: Supplemental file 1 [file 0fde6df20764e0a8cb422344ee6d8abb_MCB.00364-18-s0001.pdf]

Supplemental Materials for

Both a unique motif at the C terminus and N-terminal HEAT repeat contribute to G4 binding and origin regulation by Rif1 protein

Authors:
Shunsuke Kobayashi[1,2], Rino Fukatsu[1], Yutaka Kanoh[1] , Naoko Kakusho[1], Seiji Matsumoto, Shigeru Chaen[2] and [+]Hisao Masai[1]

Affiliations:
[1]Department of Genome Medicine, Tokyo Metropolitan Institute of Medical Science, Kamikitazawa, Setagaya-ku, Tokyo 156-8506, Japan
[2]Department of Biosciences, College of Humanities and Sciences , Nihon University

Contents

Legends to Supplemental Figures S1~S9
Supplemental Figures S1~S9

Legends to Supplemental Figures

Supplemental Figure S1.
G4 binding of various Rif1 truncation polypeptides: Estimation of binding constant.
A, **B** and **C** ^{32}P-labeled T_6G_{24} oligonucleotide (0.25 pmole), heat-denatured and renatured in 50 mM KCl and 40% PEG200, was incubated in the presence of increasing amount of the polypeptide (shown above each lace), and was analyzed on 8% PAGE (1x TBE, 50 mM KCl and 40% PEG200). The graphs show quantification of the Rif1 binding to each oligonucleotide. The values were calculated by dividing the intensities (radioactivity) of the shifted bands ("complex") by the sum of the free G4-structured DNA ("Free"; excluding the fastest migrating non-G4 structured DNA) and the shifted bands. The Kd was estimated for each polypeptide by double reciprocal plot of the binding data.

Supplemental Figure S2.
Additional set of data for Figure 3.
Additional gel shift assay data for Figure 3 are presented.

Supplemental Figure S3.
Pull-down assays of G4 binding activity of the N-terminal polypeptides of Rif1.
Purified aa1-1023 or aa1-444 polypeptides were used for pull-down assays with biotinylated G4 or non-G4 oligonucleotides (10 pmole). Non-biotinylated oligonucleotides (100 pmole) were added as competitors as indicated (lanes 3, 4, 8, and 9). A representative of at least three independent experiments are shown.

Supplemental Figure S4
Pull down assays of Rif1 truncated polypeptides (derived from the N-terminal segment) with biotin-labeled oligonucleotides
One hundred ng each of 1-444 (2 pmole) or 1-1023 (0.9 pmole) polypeptide was mixed with 10 pmole of biotinylated T_6G_{24} or $T_6(GA)_{12}$ oligonucleotide, heat-denatured and renatured, and pulled down with Streptavidin Dynabeads in the presence or absence of cold competitor DNA (50 pmole), and pulled down proteins were analyzed on 4-20% gradient gel, followed by silver staining. 5 ng of the input proteins were also run in parallel (lanes 1 and 6), which correspond to 30% and 20%, respectively, of the starting sample for the pull down.

Supplemental Figure S5.
Additional set of data for Figure 4B.
Additional gel shift assay data for Figure 4B are presented.

Supplemental Figure S6.
Additional set of data for Figure 5B.
Additional gel shift assay data for Figure 5B are presented.

Supplemental Figure S7.

Gel filtration analyses of the wild-type (wt) and mut2 Rif1 polypeptides (aa1129-1400).
The anti-FLAG column-purified Rif1 C-terminal polypeptides (aa1129-1400), wt (**A**) and mut2 (**B**) mutant, were loaded on Suprec01 column by SMART. Each fraction was analyzed on 4-20% gradient SDS-PAGE and polypeptides were detected by silver staining. Peaks of eluted polypeptides and location of each marker (analyzed in the identical manner; **C**) are marked by red arrowheads.

Supplemental Figure S8.
Strategies for construction of tagged truncation mutants of *rif1* and for isolation of *rif1* mutants that bypass Hsk1 function.
A. Rif1 was disrupted by the insertion of $Ura4^+$ gene. **B.** The Rif1 coding frame was mutated and amplified by PCR in the presence of 80 μM $MnCl_2$. **C**. A DNA fragment containing a truncated or mutated Rif1 coding frame was generated by inverse-PCR and In-Fusion-mediated joining, followed by restriction enzyme digestion and isolation of the mutated DNA fragment. **D.** DNA fragments carrying randomly mutated *rif1* (from **B**) or truncated/ point mutant *rif1* (from **C**) coding frames were transformed into *hsk1-89 rif1*Δ cells. **E.** The cells carrying mutated *rif1* were screened on 5FOA plate at 25°C (for truncation/ point mutants) or at 30ºC (for screening of bypass mutants), since replacement of $Ura4^+$ results in resistance to 5-FOA and the ability to bypass leads to growth at 30°C, the non-permissive temperature for *hsk1-89*.

Supplemental Figure S9.
Bypass mutants of Cdc7 (Hsk1) kinase isolated by random mutagenesis.
Ten-fold serial dilutions of exponentially growing *rif1* mutant cells, isolated by genetic screening as described in **Supplemental Fig. S8**, were spotted onto YES agar and incubated for 5 days at 25 or 30 ºC. The identified mutation sites in each mutant are shown.

A

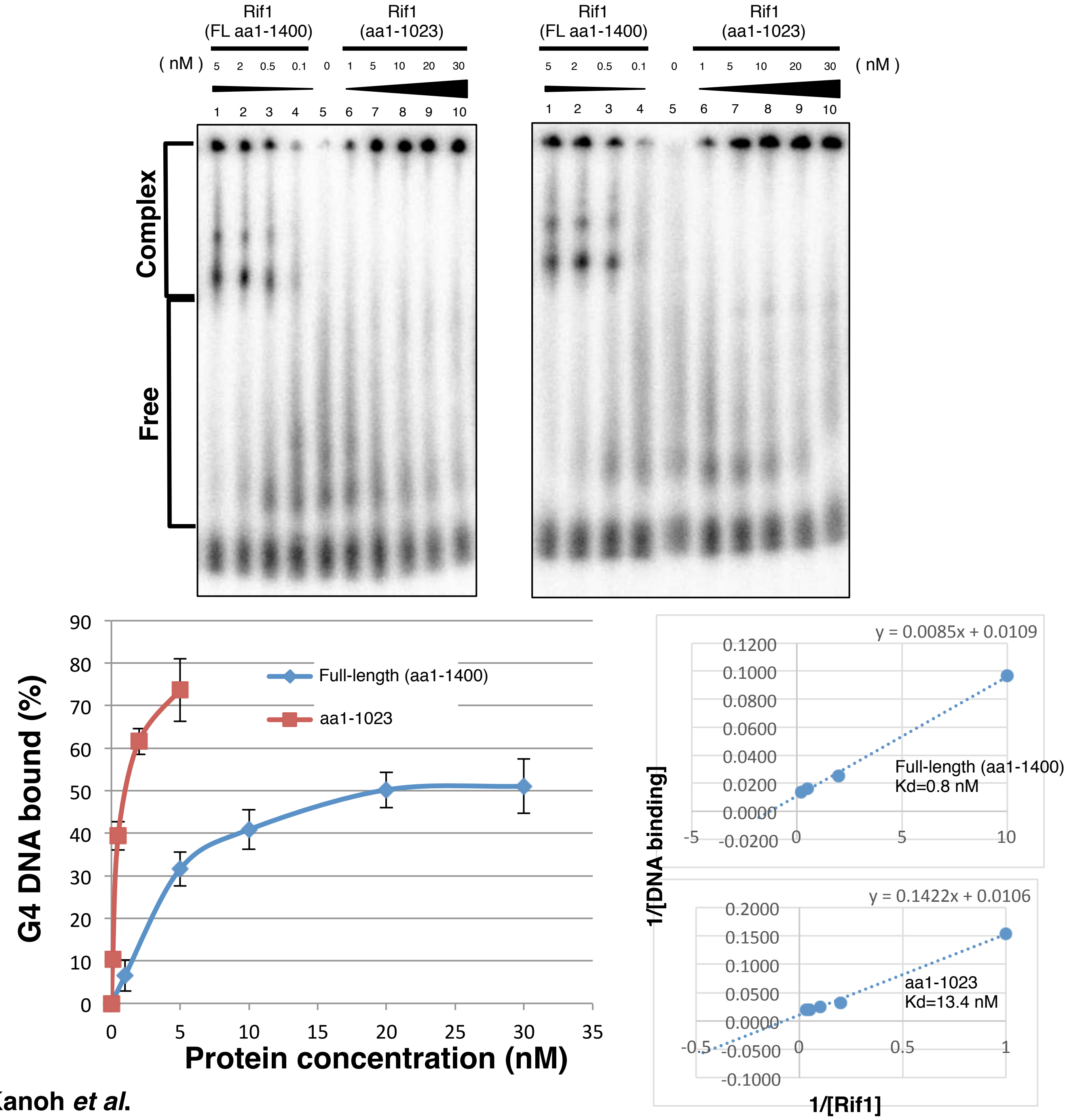

Kobayashi, Fukatsu, Kanoh *et al.*
Supplementary Figure S1

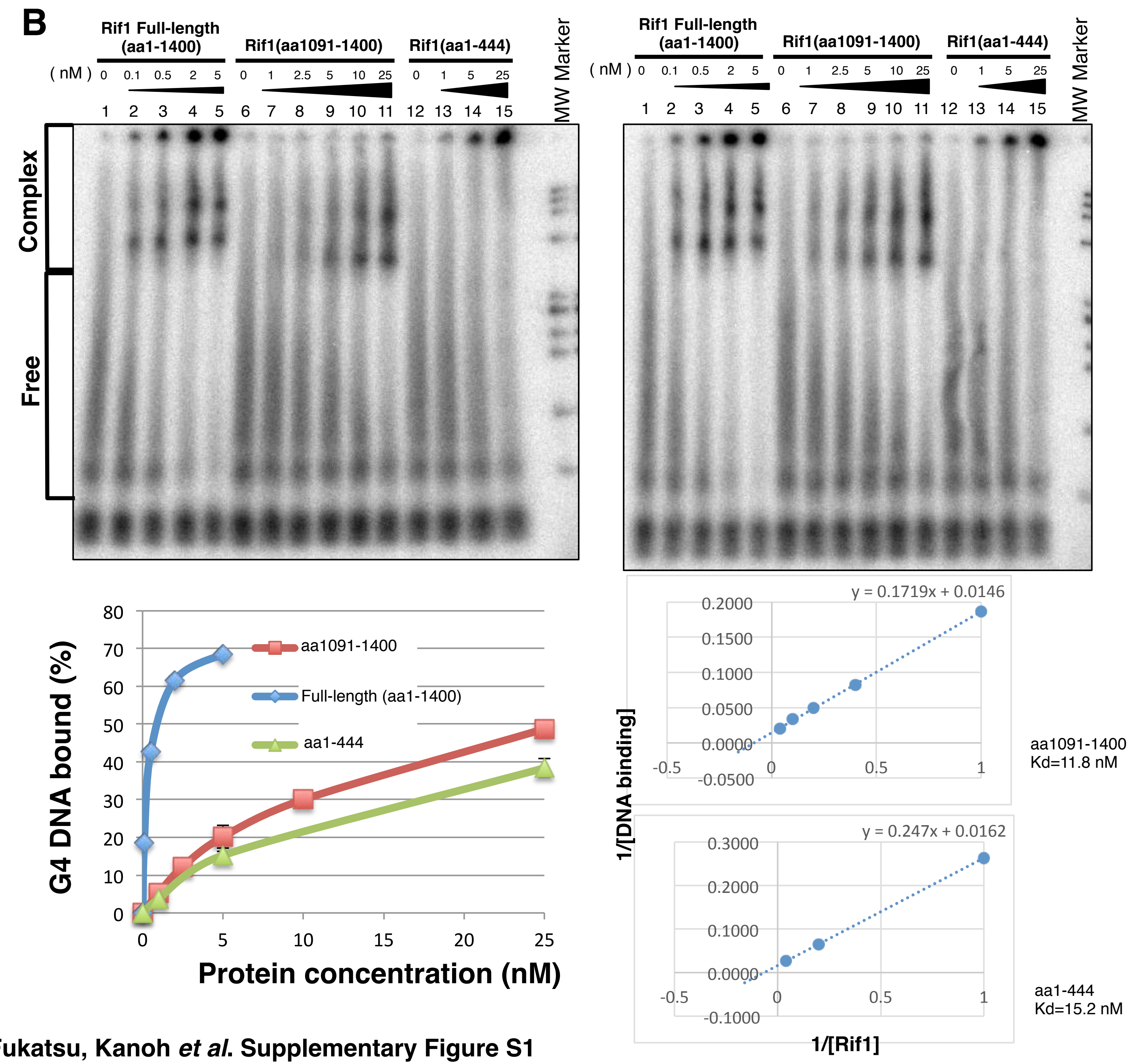

Kobayashi, Fukatsu, Kanoh *et al*. Supplementary Figure S1

C

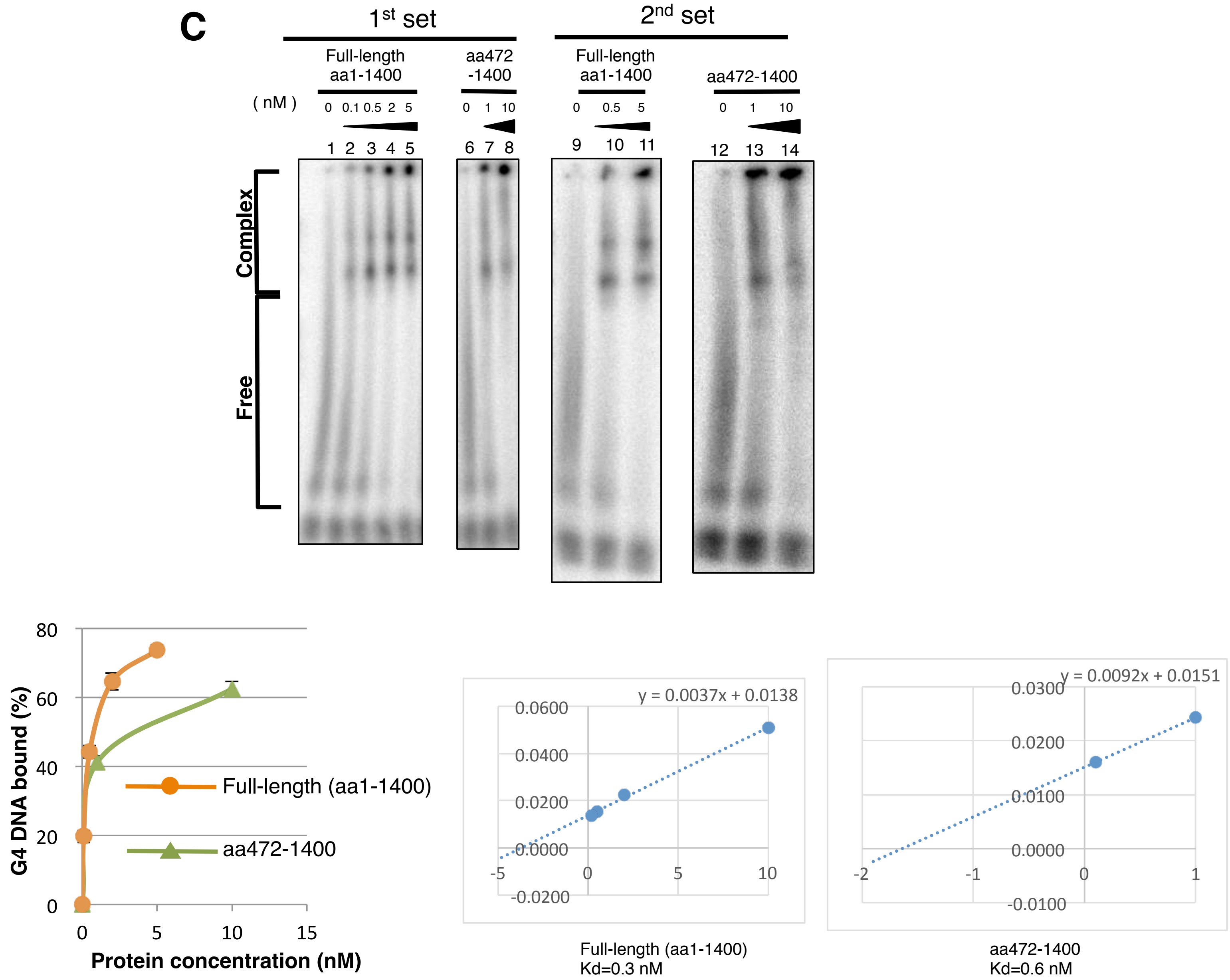

Kobayashi, Fukatsu, Kanoh *et al*. Supplementary Figure S1

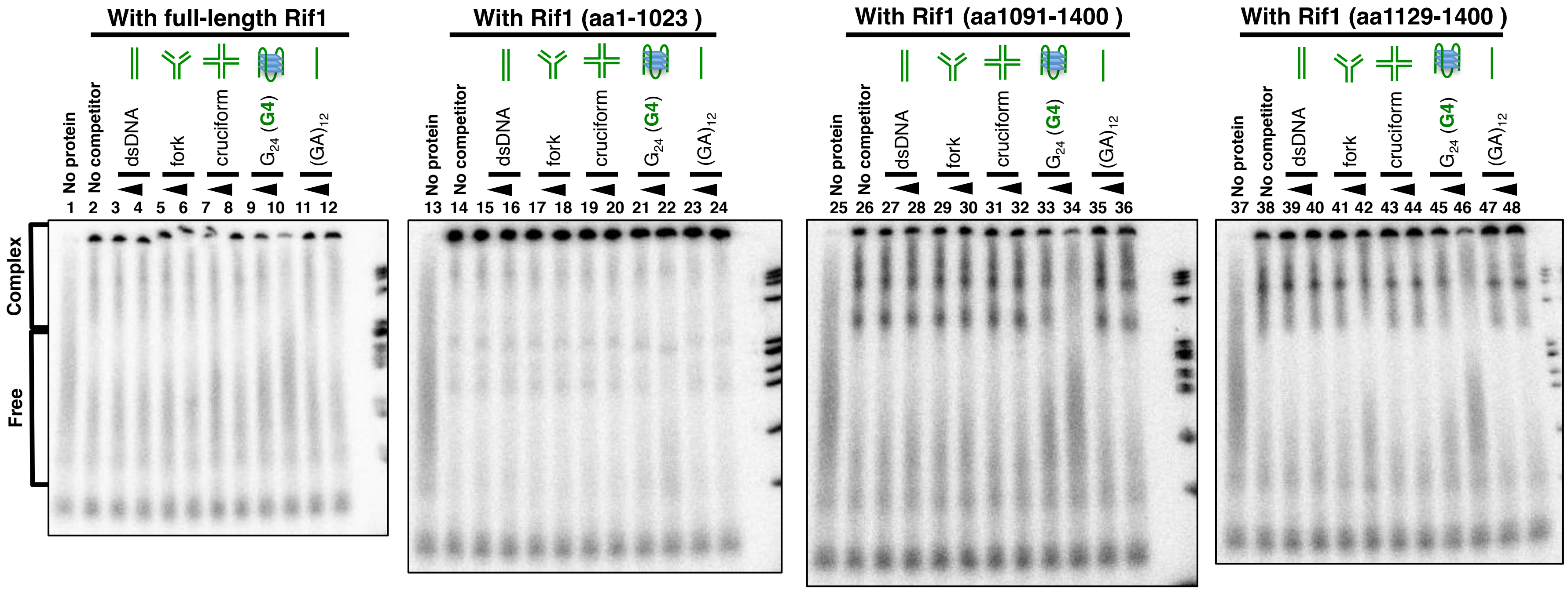

Kobayashi, Fukatsu, Kanoh *et al*. Supplementary Figure S2

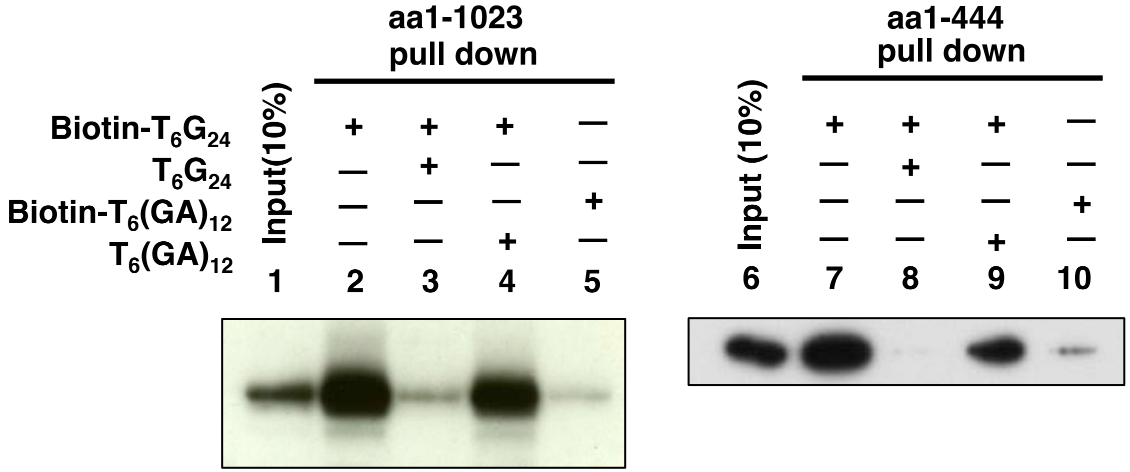

Kobayashi, Fukatsu, Kanoh *et al*. Supplemental Figure S3

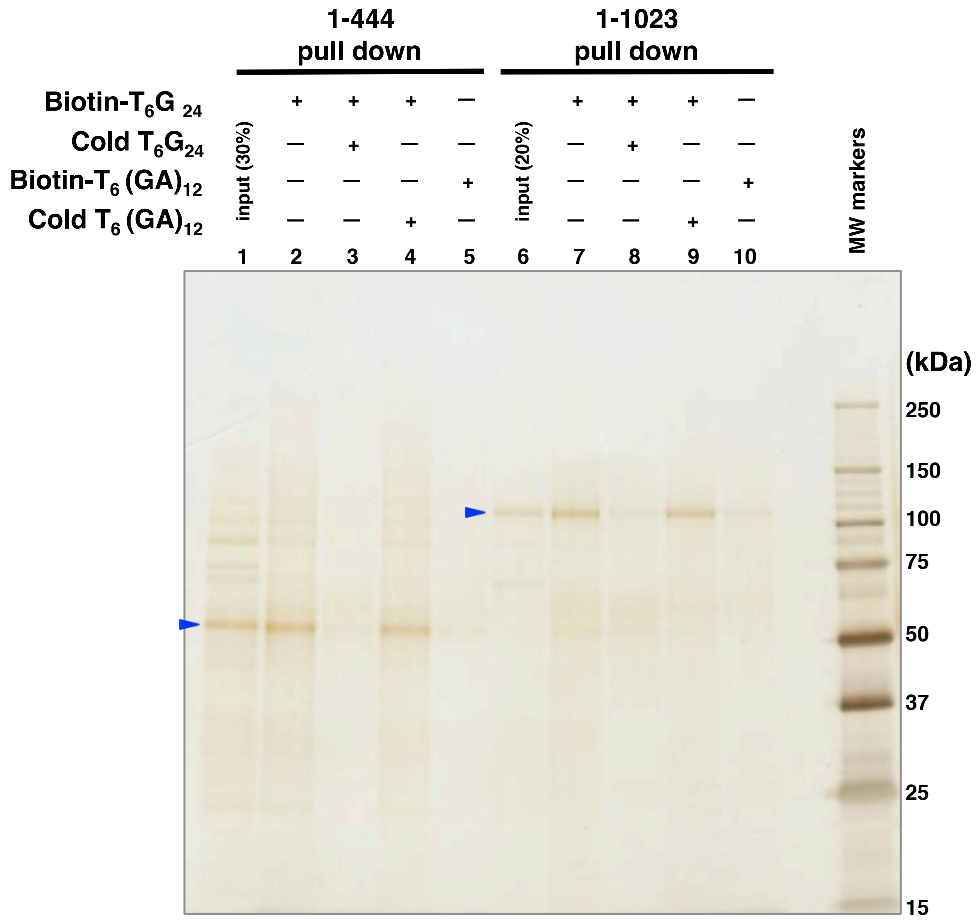

Kobayashi, Fukatsu, Kanoh *et al*. Supplemental Figure S4

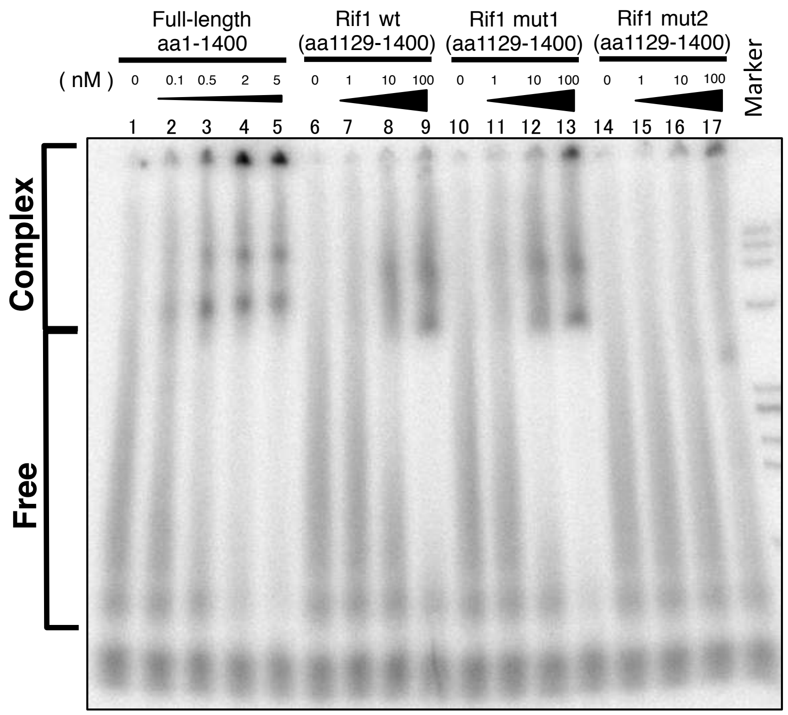

Kobayashi, Fukatsu, Kanoh *et al.*
Supplemental Figure S5

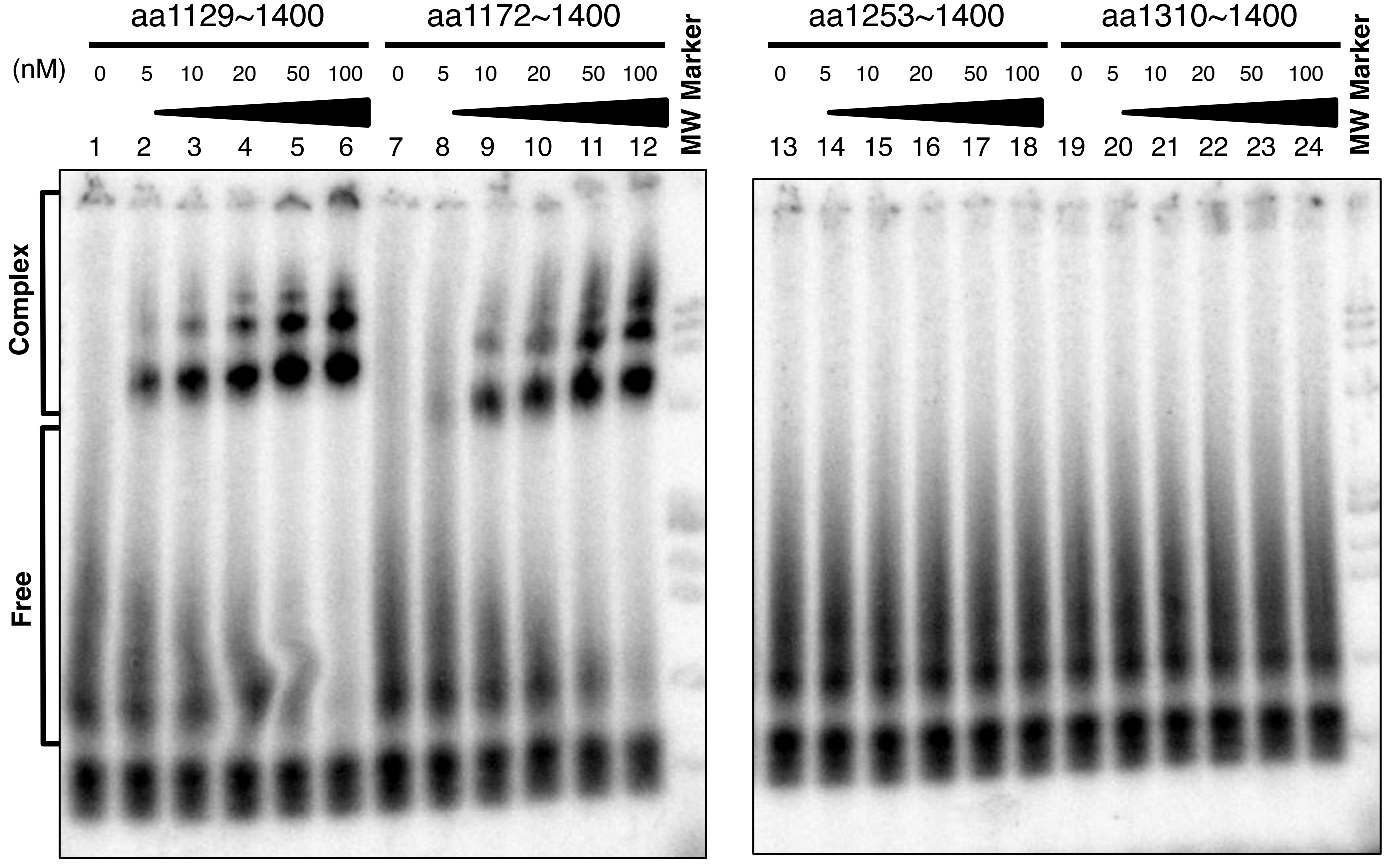

Kobayashi, Fukatsu, Kanoh *et al*. Supplementary Figure S6

A **Rif1 (aa1129-1400) wt**

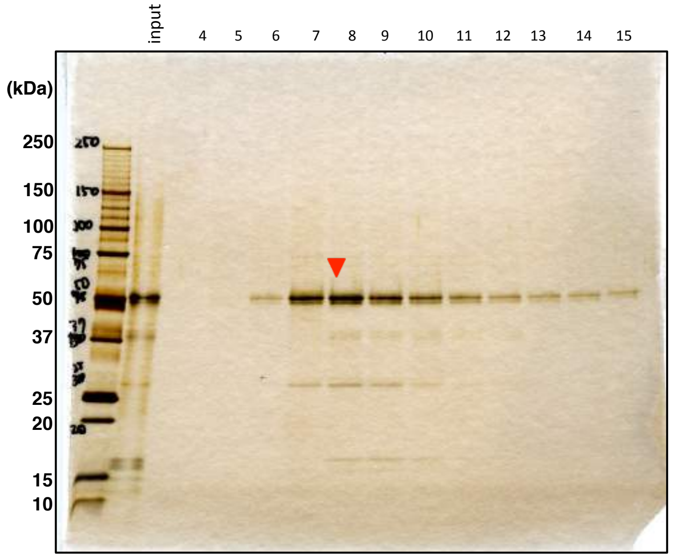

5-20% gradient gel

B **Rif1 (aa1129-1400) mut2**

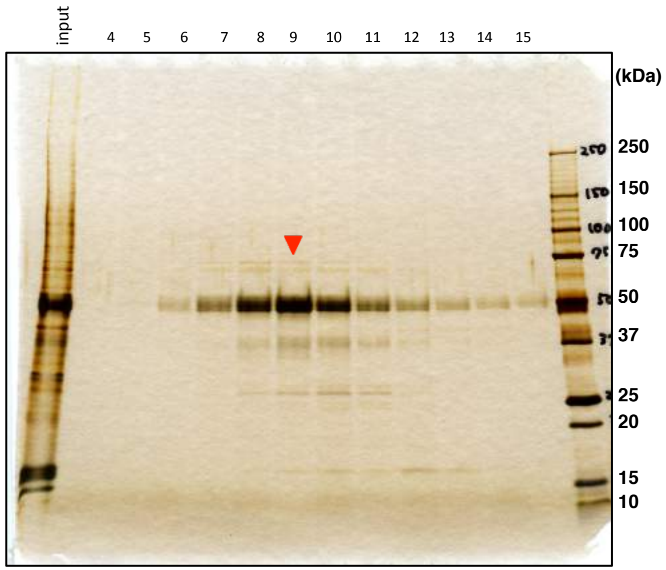

5-20% gradient gel

C **Molecular weight markers**

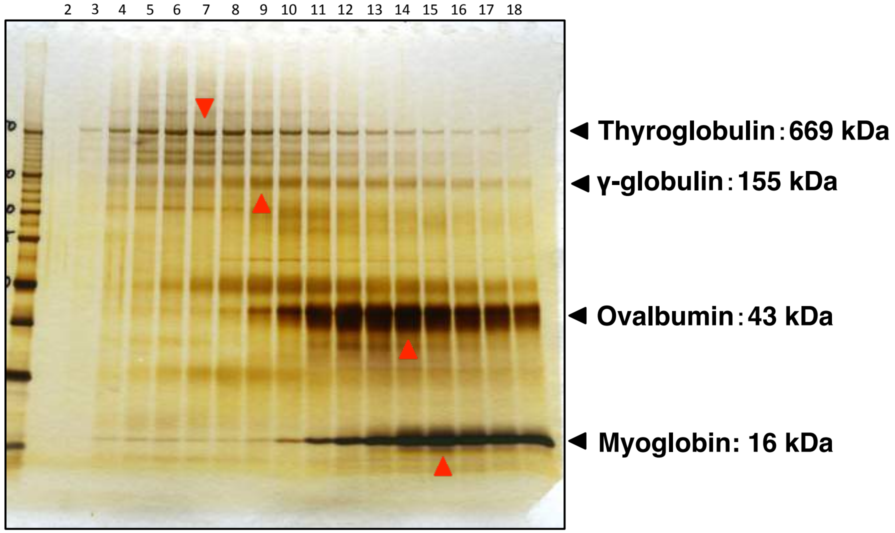

5-20% gradient gel

Kobayashi, Fukatsu, Kanoh *et al*. Supplemental Figure S7

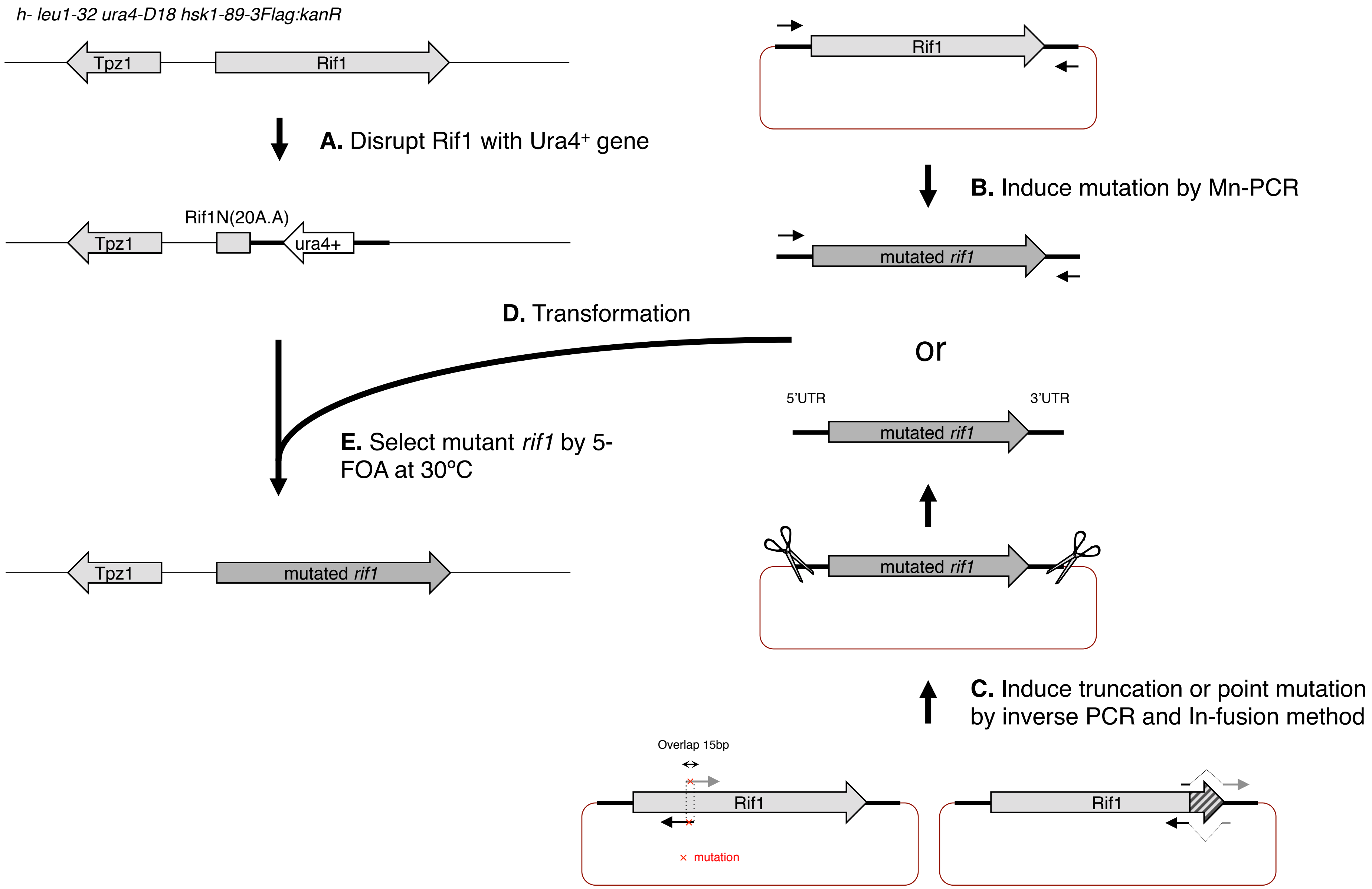

Kobayashi, Fukatsu, Kanoh *et al*. Supplementary Figure S8

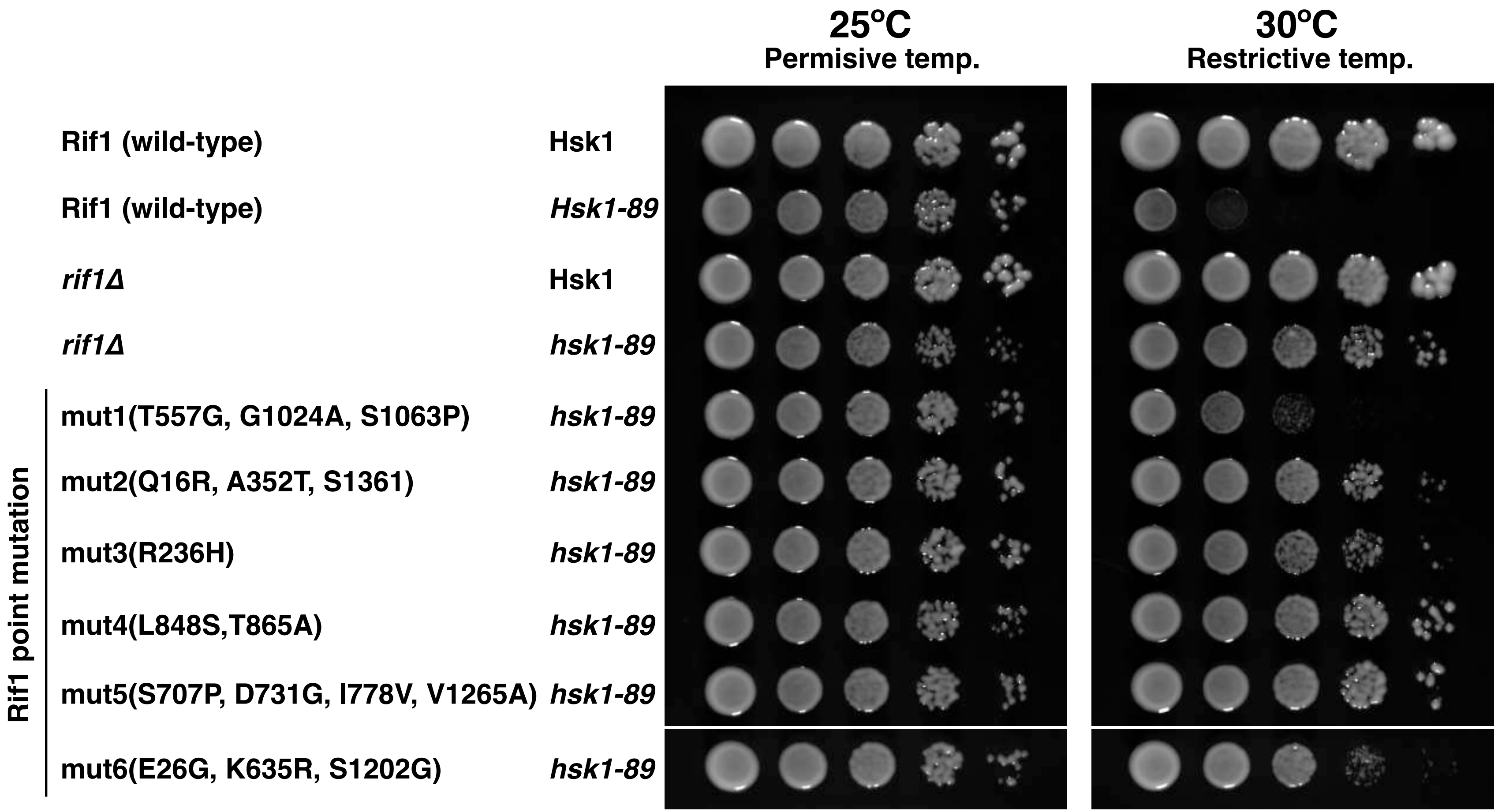

Kobayashi, Fukatsu, Kanoh *et al*. Supplementary Figure S9
